# Emerging photodynamic/sonodynamic therapies for urological cancers: progress and challenges

**DOI:** 10.1186/s12951-022-01637-w

**Published:** 2022-10-04

**Authors:** Xiao Hu, Yu-Sen Zhang, Yi-Chao Liu, Na Wang, Xian-Tao Zeng, Ling-Ling Zhang

**Affiliations:** 1 Center for Evidence-Based and Translational Medicine, Zhongnan Hospital of Wuhan University, Wuhan, 430071 China; 2 Department of Urology, Zhongnan Hosptial of Wuhan University, Wuhan, 430071 China

**Keywords:** Nanobiotechnology, Photodynamic therapy, Sonodynamic therapy, Urological cancers, Cancer therapy, Clinical translation

## Abstract

Photodynamic therapy (PDT), and sonodynamic therapy (SDT) that developed from PDT, have been studied for decades to treat solid tumors. Compared with other deep tumors, the accessibility of urological tumors (e.g., bladder tumor and prostate tumor) makes them more suitable for PDT/SDT that requires exogenous stimulation. Due to the introduction of nanobiotechnology, emerging photo/sonosensitizers modified with different functional components and improved physicochemical properties have many outstanding advantages in cancer treatment compared with traditional photo/sonosensitizers, such as alleviating hypoxia to improve quantum yield, passive/active tumor targeting to increase drug accumulation, and combination with other therapeutic modalities (e.g., chemotherapy, immunotherapy and targeted therapy) to achieve synergistic therapy. As WST11 (TOOKAD® soluble) is currently clinically approved for the treatment of prostate cancer, emerging photo/sonosensitizers have great potential for clinical translation, which requires multidisciplinary participation and extensive clinical trials. Herein, the latest research advances of newly developed photo/sonosensitizers for the treatment of urological cancers, and the efficacy, as well as potential biological effects, are highlighted. In addition, the clinical status of PDT/SDT for urological cancers is presented, and the optimization of the photo/sonosensitizer development procedure for clinical translation is discussed.

## Background

Recently, dynamic therapies are becoming 1 of the attractive therapeutic modalities for cancer treatment because of their higher disease specificity than traditional chemotherapy, which are based on the triggering of either exogenous activators (e.g., light, ultrasound (US), magnetic field, electricity and ionizing irradiation) or endogenous chemicals such as hydrogen peroxide (H_2_O_2_) to generate numerous reactive species such as reactive oxygen species (ROS) [1]. The mechanism of action can be understood as the induction of cell apoptosis or necrosis by destroying cellular components (such as proteins, lipids or nucleic acids) [1]. Photodynamic therapy (PDT) and PDT-derived sonodynamic therapy (SDT) are two typical and most promising dynamic therapies that have been well-studied. PDT uses light as a spatial–temporal controlled external trigger to activate photosensitizers (PSs) to generate ROS and subsequently induce the death of abnormal cells [2]. In 1903, Von Tappeiner used a combination of topical eosin and white light to treat skin tumors for the first time [3]. Following that little research on the clinical therapeutic applications of PDT was performed until 1972, Diamond et al. published a landmark study that first proposed the combination of tumor-targeting and phototoxicity of porphyrins to effectively treat tumors [4]. The ROS generation mechanism in PDT has been clearly revealed. Following the absorption of light (photons), the sensitizer is transformed from its ground state (singlet state) into a relatively long-lived electronically excited state (triplet state) via a short-lived excited singlet state. The excited triplet can react with oxygen to generate ROS [5]. However, traditional photodynamic therapies face critical and insurmountable limitations such as the low tissue-penetrating depth of light and potential skin phototoxicity of PSs. In particular, most clinically approved PSs respond only to ultraviolet/visible (UV/vis) light, so several strategies have been proposed for accomplishing near infrared (NIR)-activated PDT with improved tissue penetrations [6–8]. but the achieved efficacy is still much lower for deep-seated tumors. In terms of urological cancers, WST11 (TOOKAD® soluble), a palladium-coordinated bacteriochlorophyll derivative, is currently approved in the European Union (EU), Israel and Mexico for the treatment of low-risk prostate cancer (PCa), and several potential PSs are in clinical trials.

On this basis, alternative external physical activators have been recently explored to overcome the shortcomings of traditional PDT for better disease treatment, such as SDT. SDT is a new kind of dynamic therapy that exploits ultrasound (US) instead of light to generate ROS by triggering sonosensitizers. Compared with other external stimuli, US is a mechanical wave with a high penetration depth in soft tissues, which can mediate the deposition of energy to induce various biological effects, so it has been widely explored in clinical diagnosis and treatment, such as US imaging, high-intensity focused ultrasound (HIFU) and extracorporeal shock wave lithotripsy (ESWL) [9–12]. SDT effect was first discovered in 1989 by Yumita et al., who observed the cytotoxic effects of hematoporphyrin triggered by US [13]. The next year, SDT effect of hematoporphyrin in vivo was demonstrated by the same group [14]. Inspired by these phenomena, researches on possible mechanisms, novel sonosensitizers and clinical applications of SDT continuously emerged [15–18]. Different from PDT, the ROS generation mechanism in SDT has not been well documented so far. Several reliable mechanisms such as ultrasonic cavitation effect, sonoluminescence and pyrolysis are known [18]. Compared with PDT, SDT has the advantages of high safety, deep penetration and low cost, making it a favorable non-invasive treatment for various deep-seated tumors [19]. However, most of the current sonosensitizers are derived from PSs such as porphyrin derivatives, sonosensitizers with higher ROS generation efficiency remain to be developed in the future. As we all know, it is a long process from bench to bedside. Compared with SDT, so far, more effort has been devoted to the study of PDT and a large number of clinical trials have been carried out. Therefore, although SDT is derived from PDT and has a wider range of application scenarios, it cannot completely replace PDT at present and more comprehensive evidence is needed.

Urological system organs, such as kidney, bladder and prostate, play an important role in excretion, regulation of acid–base balance and maintenance of body homeostasis. Unfortunately, urological cancers are a serious threat to the health and life of human beings [20]. According to the latest statistical data, the incidence rate of urological cancers, including kidney cancer, bladder cancer (BC) and prostate cancer (PCa), accounted for more than 12.5% of 36 cancers in 2020. In particular, the incidence rate of prostate cancer has risen to the third highest, inferior to that of female breast cancer and lung cancer [21]. Traditional therapeutic modalities of urological cancers include surgery, chemotherapy and radiotherapy. During the past decades, various novel therapeutic modalities have been discovered and implemented clinically to provide patients with improved therapeutic outcomes, including androgen deprivation therapy, immunotherapy, targeted therapy, etc. [22–26]. However, there are still some limitations of these novel therapies, including the complex nature of the molecular targets, severe side effects and high prices [27, 28]. The accessibility of urological organs makes them more suitable for therapeutic modalities like PDT/SDT, which require exogenous stimulation. Although most urological tumors (such as prostate tumor and bladder tumor) can currently be minimally invasive operated through the urethra, PDT can be performed through a similar approach with less damage than surgery. As for SDT, it can be performed externally by US, which is non-invasive. Compared with the systemic adverse reactions or complications caused by other drug treatments and radiotherapy, the high spatial–temporal controllability of PDT/SDT makes it an alternative to these commonly used clinical treatments. An increasing number of researches on nanobiotechnology-assisted PDT/SDT have been reported, aiming to make it a promising therapeutic modality that can complement the current therapeutic procedures for urological cancers.

Due to the complexity and heterogeneity of the tumor microenvironment (TME), the delivery of sensitizers and their responses in tumor areas remains challenging. Considering that nanomedicine has made many technological breakthroughs [29], it has been applied to PDT/SDT in order to improve the efficacy from the following aspects. First, the pharmaceutical properties of the drug (e.g., stability, solubility, circulating half-life) can be improved to improve bioavailability. Second, nanoparticles with active targeting properties can be designed to increase the accumulation of sensitizers in tumor areas. Third, the nanoparticles can be designed with controllable switches to preserve the photochemical/sonochemical property of the sensitizer before the reaction. Fourth, nanoparticles can enhance energy transfer and improve ROS generation efficiency. Fifth, nanoparticles can be utilized to combine PDT/SDT with other proven therapeutic modalities (e.g., chemotherapy, immunotherapy, targeted therapy and so on). Last but not least, nanoparticles can remain highly biocompatible to ensure further clinical translation [1, 30, 31].

**Fig. 1:**
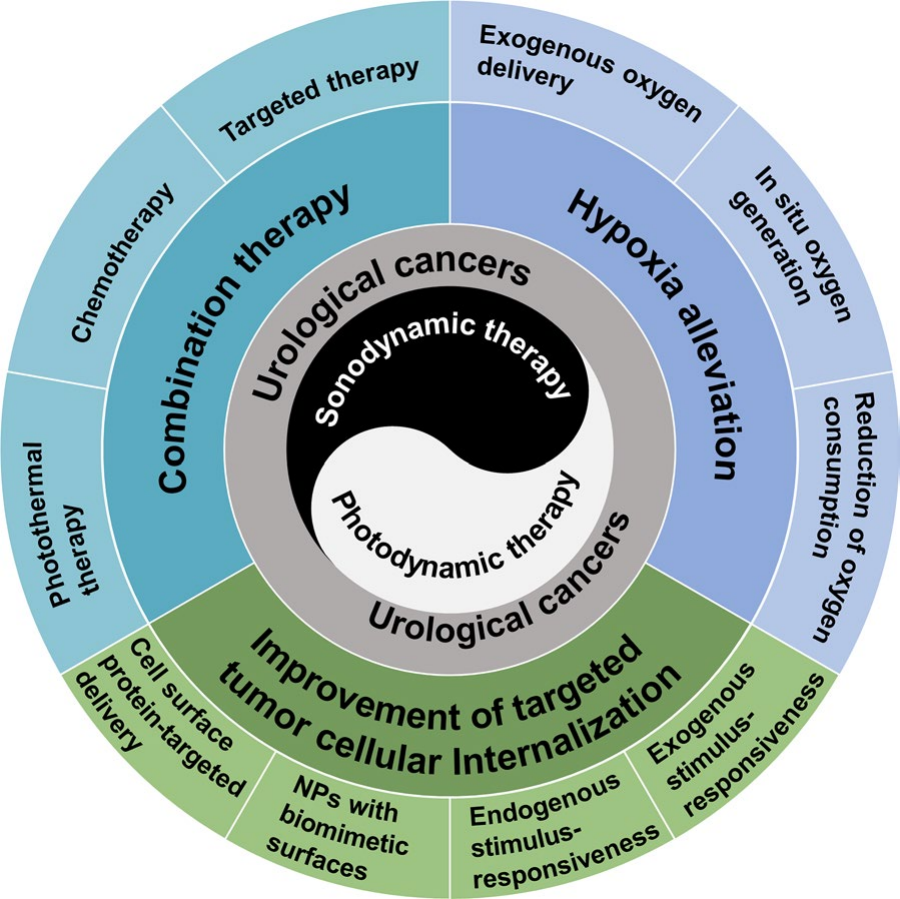
Newly developed strategies of enhanced PDT/SDT for urological cancers

In this review, we illustrate the design principles and therapeutic potential of novel PDT/SDT for urological cancers in preclinical models by introducing representative paradigms of the past 10 years (Fig. 1), and then analyze the clinical trials that have been conducted to date to gain insight into its challenges and prospects in clinical translation.

## The latest advances in preclinical researches of PDT/SDT for urological cancers

Preclinical cancer research is carried out on appropriate tumor models (such as cell lines and animal models) that simulate the human in vivo environment to evaluate the efficacy and safety of the treatment before clinical trial (experiment on human beings). To advance PDT/SDT for urological cancers from bench to bedside, more high-quality preclinical researches are required. Herein, the last decade of preclinical studies of PDT/SDT with proven efficacy both in vitro (cells) and in vivo (experimental animals) are presented.

### PDT for urological cancers

As mentioned above, PDT is a non-invasive therapeutic modality that works through the combined reaction of light, PSs and oxygen molecules in tumor tissues. Early PDT researches were mostly based on the first-generation PSs (e.g., hematoporphyrin derivatives) or second-generation PSs (e.g., 5-aminolevulinic acid, chlorin, hematoporphyrin monomethyl ether), which suffer from poor bioavailability, poor selectivity, and low efficiency [32]. Owing to the revolutionary progress of nanotechnology in recent years [33, 34], the arsenal of PSs is expanding rapidly. Herein, these novel technologies were introduced into PDT with the aim to satisfy the following three ideal conditions. First, PSs can preferentially accumulate in tumor tissues at a specific time and have a high quantum yield of ROS. Second, a light source that matches the characteristic absorption of PSs can be timely directed to irradiate PSs in tumor tissues. Last but not least, there is sufficient oxygen in TME to react with activated PSs to generate ROS.

#### PDT for bladder cancer

BC is the 10th most commonly diagnosed cancer worldwide in 2020 statistics, with approximately 573,000 new cases and 213,000 deaths. The global incidence and mortality rates for men are 9.5 and 3.3 per 100,000 respectively, which are approximately 4 times higher than for women. Thus the disease ranks higher among men, for whom it is the 6th most common cancer and the 9th leading cause of cancer death [21]. According to the depth of invasion, BC is divided into non-muscle-invasive bladder cancer (NMIBC) (75% of new cases) and muscle-invasive bladder cancer (MIBC) or metastatic cancer (25% of new cases). NMIBC is mainly treated by transurethral resection of bladder tumor (TURBT), followed by intravesical chemotherapy or immunotherapy to prevent disease recurrence and progression. Conventional treatment for MIBC is radical cystectomy and urinary diversion, with or without adjuvant therapy [23, 24]. Despite evolving treatment strategies, the overall prognosis of BC has not improved significantly over the past four decades. NMIBC has a reported recurrence rate of 50%-90% within five years, which means new techniques are urgently needed to improve the therapeutic efficacy of BC [35]. BC is well-suited for PDT because bladder is an easily accessible and hollow organ for both intravesical instillation and illumination. In 1975, J.F. Kelly et al. first reported the application of PDT in the treatment of BC, who observed the destruction of tumors by light irradiation to the bladder after systemic administration of hematoporphyrin derivative (HPD) [36]. The following year, Kelly and Snell initiated the first human trial of PDT for BC. A patient with recurrent BC who had failed transurethral resection, radiotherapy, and chemotherapy was treated with PDT. In this case, HPD slowed tumor growth and tumor necrosis happened in the area receiving PDT [37]. These groundbreaking researches brought a new strategy to the treatment in BC. Emerging strategies of nanobiotechnology-enhanced PDT for BC over the past 10 years are summarized in Table 1.

**Table 1:** Emerging strategies of enhanced PDT for bladder cancer

Photosensitizers	Biological model	Strategy	Result	Refs.
ALA	Subcutaneous 253 J B-V in nude mice	Combination with DFX to increase PpIX accumulation	Enhancing ALA-PDT-induced cell apoptosis	[38]
HSA-MnO_2_-Ce6 NPs	Orthotopic MB-49 in C57BL/6 mice	Catalyzing the decomposition of H_2_O_2_ to generate oxygen; serving as a good contrast reagent for MRI	Improved therapeutic efficacy and prolonged lifetime of mice compared to controls	[39]
HSA-Ce6/NTZ/FCS NPs	Orthotopic fLuc-T24 in nude mice	Intravesical instillation; reduced tumoral oxygen consumption; enhanced transmucosal ability	Dramatically enhanced orthotopic bladder tumors ablation	[40]
PorGal_8_	Subcutaneous UM-UC-3luc^+^ in nude mice	Targeting galectin-1	PDT-mediated tumor shrinkage and downregulation of E-cadherin	[41]
ChlGal_8_	Subcutaneous HT-1376 in nude mice	Targeting galectin-1; better photochemical and photophysical properties	Improved efficacy after repeated PDT in resistant cells	[42]
PLZ4-nanoporphyrin	Orthotopic and subcutaneous PDX in NSG mice	Targeting α_V_β_3_ integrin; combination with PTT and chemotherapy	Orthotopic PDX bladder tumors elimination after intravesical light irradiation	[43]
Gd-PEG-R^3^	Subcutaneous T24 in nude mice	Targeting α_V_β_3_ integrin; “off–on” responsive theranostic agents	Selective bladder tumor suppression; enhanced in vivo MRI signal upon binding	[44]
Porphyrin-DNA NPs	Subcutaneous 5637 in nude mice	Hydrophobicity-dependent DNA and PSs release	Light-dependent tumor suppression	[45]
TNP/DOX/ZnPC	5637 in nude mice	Combination with DOX-based chemotherapy	Higher tumor suppression compared to PDT alone or chemotherapy alone	[46]
BITT@BSA-DSP NPs	Subcutaneous MB49 in C57BL/6 mice	Combination with PTT, chemotherapy and AIEgens-based NIRFI; reduction-responsive drug delivery	Efficient visualization of tumor regions and significant tumor suppression	[47]
Ppa-hydrazone-DOX NPs	Subcutaneous PDX in NSG mice	Combination with PTT and chemotherapy to overcome drug resistance; pH-responsive drug delivery	Eradication of DOX- and GDC-resistance tumors	[48]
Poly (OEGMA)-PTX prodrug@Ce6	Subcutaneous PDX in nude mice	Combination with PCI and chemotherapy; enzyme-responsive drug delivery	Complete tumor eradication after two-stage irradiation	[49]
17-AAG-loaded nanoporphyrin	Subcutaneous PDX in NSG mice	Combination with NIRFI, PTT and HSP90-targeted therapy	Superior anti-cancer efficacy with downregulation of HIF-1α, Akt, Erk and Src, and upregulation of HSP70	[50]
Panitumumab-IR700 conjugates	Subcutaneous UM-UC-5 and UM-UC-3 in nude mice	EGFR-targeted NIR-PIT	EGFR surface expression-dependent tumor suppression by cell necrosis	[51]
Erlotinib-PS	Subcutaneous UM-UC-3 and T24 in SCID mice	EGFR-targeted PIT; combination with PET imaging for fluorescence-guided PDT	Long-term cure for EGFR-positive tumors; PET imaging ability	[52]
Pan-IR700 + tra-IR700	Subcutaneous SW780 in nude mice	Combination of EGFR- and HER2-targeted NIR-PIT	Strongest tumor suppression compared to either agent alone	[53]
Anti-CD47-IR700	Subcutaneous GFP-luciferase transfected 639 V in NSG mice	CD47-targeted NIR-PIT	Prominent tumor suppression and prolonged survival by 5 rounds of treatment	[54]

*ALA 5*-Aminolevulinic acid; *DFX* Deferoxamine; *PpIX* Protoporphyrin IX; *NP* Nanoparticle; *PS* photosensitizer; *HSA* Human serum albumin; *Ce6* Chlorin e6; *MRI* Magnetic resonance imaging; *NTZ* Nitazoxanide; *FCS* Fluorinated chitosan; *PDX* Patient-derived xenograft; *NSG* NOD scid gamma; *PTT* Photothermal therapy; *PEG* Polyethylene glycol; *TNP* Thermal-responsive nanoparticle; *DOX* Doxorubicin; *ZnPC* Zinc phthalocyanine; *BSA* Bovine serum albumin; *AIEgens* Aggregation-induced emission luminogens; *NIRFI* Near-infrared fluorescence imaging; *Ppa* Pheophorbide a; *GDC* GDC-0941; *PTX* Paclitaxel; *PCI* Photochemical internalization; *17-AAG* 17-allylamino-17-demethoxygeldanamycin; *HSP* Heat shock protein; *HIF-1α* Hypoxia-induced factor 1α; *Akt* protein kinase B; *Erk* Extracellular signal-regulated kinase; *IR700* IRDye700Dx; *EGFR* Epidermal growth factor receptor; *NIR* Near-infrared; *PIT* Photoimmunotherapy; *SCID* Severe combined immunodeficient; *PET* Positron emission tomography; *pan* Panitumumab; *tra* Trastuzumab

##### Enhanced PDT in combination with clinically approved drugs

Boosting the efficacy of PDT by taking clinically approved drugs may be a simple and easy strategy. Photodiagnosis (PD) and photodynamic therapy (PDT) mediated by 5-aminolevulinic acid (ALA) have been investigated for decades [55]. It has been reported that ALA can induce the tumor-selective accumulation of the photosensitizer protoporphyrin IX (PpIX) in BC cells [56, 57]. Inoue et al. found that deferoxamine (DFX), a ferrochelatase inhibitor, could inhibit the metabolism of PpIX to heme in BC cells in a time- and concentration-dependent manner, thereby increasing the accumulation of PpIX and ultimately enhancing ALA-PDT-induced cell apoptosis [38].

##### Hypoxia alleviation

The rapid tumor progression increases the demand for blood in the tumor tissue, which causes vascular disorder and insufficient blood supply inside the tumor and ultimately makes the tumor cells hypoxia [58]. Several researchers have described the TME of BC is hypoxic [59–61], which limits the antitumor effect of PDT because the generation of ROS requires sufficient oxygen molecules to participate in the reaction. Furthermore, the continuous depletion of oxygen during PDT aggravates tumor hypoxia again. Therefore, alleviating tumor hypoxia is 1 of the key methods to improve the efficacy of PDT. There are three main strategies to design nanoparticles to overcome tumor hypoxia, such as exogenous oxygen delivery, in situ oxygen generation and reduction of oxygen consumption [62].

###### In situ oxygen generation

**Fig. 2:**
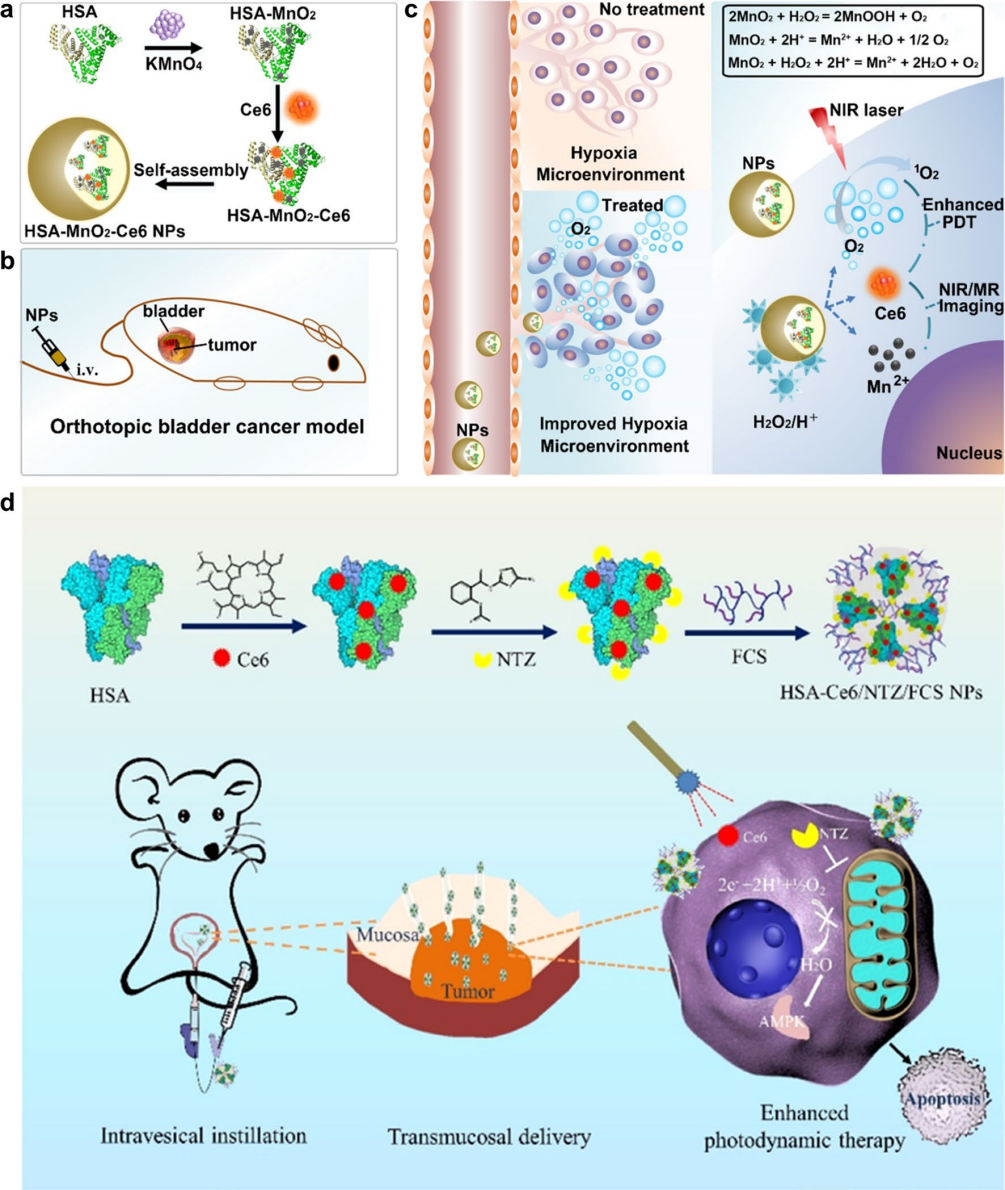
**a-c** Schematic illustrations of the synthesis of HSA-MnO_2_-Ce6 NPs (**a**) and NPs-mediated enhanced PDT for orthotopic BC (**b**) by alleviating hypoxia (**c**). Reprinted with permission [39]. Distributed under a Creative Commons Attribution (CC BY-NC 4.0) license. **d** Schematic illustration of the synthesis of HSA-Ce6/NTZ/FCS NPs and enhanced intravesical PDT for orthotopic BC by transmucosal NPs delivery. Reprinted with permission [40]. Copyright 2021, American Chemical Society

Hypoxia-induced metabolic shift in BC cells also produces higher amounts of H_2_O_2_ and leads to acidosis [63, 64]. Catalase (CAT) can catalyze the decomposition of endogenous H_2_O_2_ overexpressed in tumor tissues to generate oxygen. In addition, many metal-based nanozymes including Pt [65], Mn [39], prussian blue (PB) [66] and Fe [67] based nanozymes possess CAT-like properties. MnO_2_ can catalyze the decomposition of H_2_O_2_ to generate oxygen and itself also decomposes under acidic conditions to release Mn^2+^ ions that serve as a good contrast reagent for magnetic resonance imaging (MRI) [68, 69]. Lin et al. fabricated HSA-MnO_2_-Ce6 NPs by self-assembly, consisting of MnO_2_, Ce6 (chlorin e6, a NIR-activated second-generation PS with high quantum yield) and HSA (human serum albumin, a famous drug carrier protein with good biocompatibility) (Fig. 2a) [39]. In vivo study on an orthotopic BC mouse model (Fig. 2b), which can more closely simulate the natural TME and presence of supporting cells, a large amount of oxygen was in situ generated in tumor tissues after systemic intravenous injection of HSA-MnO_2_-Ce6 NPs (Fig. 2c), and more complete tumor ablation and longer lifespan in mice than controls was observed after PDT.

###### Reduction of oxygen consumption

Intravesical instillation therapy has been an indispensable approach for the treatment of BC to avoid systemic toxic side effects of intravenous administration [70]. Therefore, intravesical PDT for BC has potential to improve local PSs concentrations and alleviate skin phototoxicity. Wu and coworkers previously reported that fluorinated chitosan (FCS) could be utilized for intravesical instillation to efficiently transmucosal transport proteins by reversibly modulating the tight junctions of the bladder epithelium [71]. Therefore, they developed a 2-step self-assembly approach to form a kind of transmucosal nanophotosensitizers named HSA-Ce6/NTZ/FCS NPs (HSA for human serum albumin, NTZ for nitazoxanide) by simply mixing HSA-Ce6 with NTZ and FCS sequentially (Fig. 2d) [40]. Herein, HSA was used to improve biocompatibility, and NTZ, an FDA-approved anti-helminth drug that has been shown to be an effective regulator for mitochondrial respiration and crucial metabolic signaling [72, 73], was used to alleviate tumor hypoxia for enhanced PDT. Prominently improved PDT efficacy was achieved after intravesical instillation of HSA-Ce6/NTZ/FCS NPs in an orthotopic bladder tumor mouse model, providing a promising strategy for clinical intravesical PDT.

Several kinds of PSs have been approved for clinical use [5]. Unfortunately, they have poor tumor selectivity, which may cause unwanted side effects and damage to healthy tissues [74]. However, traditional PSs can be skillfully modified to enable passive or active tumor targeting.

##### Improvement of targeted tumor cellular internalization

Targeted drug delivery to tumor tissue has long been a critical issue in medicine [75], which consists of two parts, one is to increase drug accumulation in tumors and the other is to reduce non-specific distribution of drugs. Modification of PSs (or sonosensitizers) with targeting peptides to target proteins overexpressed on the surface of tumor cells is one of the most straightforward strategies [41–44]. In addition, nanoparticles endowed with the ability to release drugs in response to endogenous or exogenous stimulus through nanobiotechnology have also been employed to increase drug accumulation in tumor tissue [76–80]. Recently, imparting a biomimetic coating to the surface of nanoparticles has gradually proven to be a promising approach to achieve targeted drug delivery [81, 82].

###### Cell surface protein-targeted delivery

**Fig.3:**
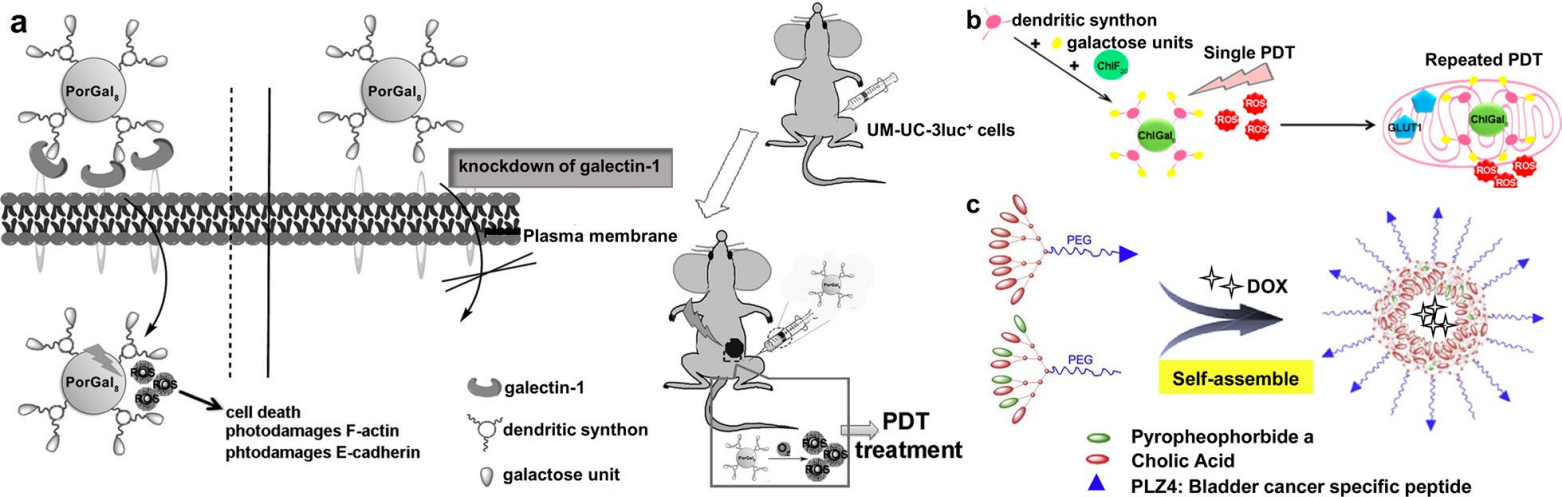
**a** Schematic illustration of the phototoxicity of PorGal_8_ in vitro and in vivo biological models containing high levels of galectin-1 protein. Reprinted with permission [41]. Copyright 2016, Elsevier. **b** Schematic illustration of the design of ChlGal_8_ and the ability to accumulate in the mitochondria by facilitative GLUT1 after repeated PDT. Reprinted with permission [42]. Copyright 2016, American Chemical Society. **c** Schematic illustration of self-assembly of PLZ4-nanoporphyrins and their functional components. Reprinted with permission [43]. Copyright 2016, Elsevier

Galectin-1 protein is known to be overexpressed in many tumor tissues (e.g., bladder tumors) [83, 84]. Based on the understanding of that, Pereira et al. developed a targeted porphyrin, PorGal_8_, conjugated with dendritic units of galactose that has a high affinity to galectin-1 proteins [41]. They demonstrated the ability of PorGal_8_ to preferentially accumulate in tumor tissues in a xenograft bladder tumor model, which includes mice with UM-UC-3 cells (containing high levels of galectin-1) (Fig. 3a). They also reported another new third-generation PS, a chlorin conjugated with galactodendritic units namely ChlGal_8_, with better photochemical and photophysical properties than PorGal_8_. Owing to the ability to accumulate in the mitochondria, via facilitative glucose transporter 1 (GLUT1), in the period between single and repeated irradiation, ChlGal_8_ can efficiently enhance the phototoxicity in PDT-resistant HT-1376 cells after repeated PDT (Fig. 3b) [42].

α_V_β_3_ integrins are composed of dimeric non-covalently bound transmembrane alpha-v and beta-3 (α_V_β_3_) sub-units [85], functioning as receptors for extracellular matrix proteins to regulate the migration and survival of normal and tumor cells [86]. Known that α_V_β_3_ integrins are generally low expressed on epithelial cells and mature endothelial cells in normal tissues, but highly expressed on the surface of neovascular endothelial cells in bladder tumors [87], they have become promising targets for the treatment of BC. Lin et al. performed a high-throughput one-bead one-compound combinatorial chemistry approach to identify a cyclic peptide named PLZ4 (amino acid sequence: cQDGRMGFc) that could selectively bind to the α_V_β_3_ integrin on BC cells [88, 89]. They modified this BC-specific ligand onto the surface of micelles loaded with the chemotherapeutic drug paclitaxel, demonstrating that targeted micelles showed superior antitumor efficacy in comparison to non-targeted drug-loaded micelles and free drug both in vitro and in vivo [90]. Therefore, they designed a BC-targeted nanoparticle platform called PLZ4-nanoporphyrin (PNP) for PDT, which consists of a newly introduced porphyrin-cholic acid (CA)-polyethylene glycol (PEG) conjugate (PEG^5k^-Por_4_-CA_4,_ providing photodynamic diagnosis/therapy) and an original targeted ligand-cholic acid-polyethylene glycol conjugate (PLZ4-PEG^5k^-CA_8,_ providing molecular targeting) (Fig. 3c) [43]. After loading with doxorubicin (DOX), this novel PNP-DOX platform exhibited excellent antitumor efficacy in an orthotopic patient-derived xenograft (PDX) BC mouse model due to the integration of 3 therapeutic modalities, namely photodynamic/photothermal/chemotherapy.

It is known that PDX models are valuable tools for preclinical drug testing. In PDX models, cancer specimens that were surgically removed from patients are transplanted in mice to better mimic tumor heterogeneity and architecture [91]. Therefore, researches on a PDX model have greater potential for clinical translation.

Three α_V_β_3_-integrin-specific peptides, R^1^ (amino acid sequence: cQDGRMGFc), R^2^ (cGRLKEKKc), and R^3^ (RrRKcGRLKEKKc), were obtained by a combinatorial chemistry approach. Conjugated with these three peptides, respectively, lanthanide-porphyrinato complexes can selectively bind to receptors on the membrane of BC cells with no invasion to normal cells [92]. The relatively hydrophobic R^2^ was conjugated with a hydrophilic peptide RrRK, resulting in an amphiphilic R^3^ with an improved cell membrane permeability. Based on this earlier report, Xie et al. synthesized a porphyrinato-gadolinium complex Gd-PEG-R^3^, introducing PEG linker for better biocompatibility [44]. The Gd-PEG-R^3^ complex, showed high efficiency of PDT to selectively kill BC cells at the cellular level and inhibit bladder tumors in xenograft-bearing nude mice, and enhanced the in vivo “off–on” MRI signal for its low initial T_1_ relaxivity increasing over 17 times upon α_V_β_3_ binding. Moreover, multifunctional nanoplatforms can be well-designed to combine PDT with other therapeutic modalities for synergistic therapy.

##### Combination therapy

As good drug carriers, multifunctional nanoparticles can rationally combine PDT (or SDT) with other therapeutic modalities to improve efficacy. Ghosh et al. synthesized a kind of nanoparticles with hydrophobicity-dependent DNA release and photodynamic antitumor activity through non-covalent assembly of meso-tetra-4-pyridyl porphine (MTP) with single-stranded DNA (ssDNA) [45]. These porphyrin-DNA nanoparticles (PDN) are stable in aqueous solution under physiological conditions and can dissociate upon cellular internalization because of the hydrophobic environment of cell membrane, which is important for the delivery of photosensitizers and DNA-based therapeutic payloads. Both light-dependent cytotoxicity in vitro and antitumor activity towards BC xenografts in vivo of PDN were demonstrated.

It is noted that the most feasible combination therapies are based on combining PDT (or SDT) with clinically mature drugs (such as chemotherapy drugs [46–49], inhibitors of specific targets [50–54]). In addition, since photothermal therapy (PTT) also employs light as an activator, it has also been combined with PDT in some studies [93].

###### Combination with chemotherapy

Neoadjuvant combination chemotherapy (NAC) with cisplatin-based combinations is the standard of care for patients with resectable invasive BC. However, only cisplatin-fit patients are candidates [23]. PI3K inhibition could sensitize drug-resistant BC cells to cisplatin and gemcitabine treatment in vitro and in vivo. But the development of secondary resistance toward the PI3K inhibitor ultimately led to treatment failure like all other targeted therapy [94]. Therefore, it’s urgent to develop a therapeutic strategy to overcome the resistance to both chemotherapy and targeted therapy. PDT has been used in combination with chemotherapy, anti-angiogenesis therapy, and targeted therapy because it is capable of alleviating the co-activation and compensation of drug-resistance-related molecular signaling pathways [95–97]. Huang et al. validated the feasibility of combination therapy by combining zinc phthalocyanine (ZnPC)-based PDT with DOX-based chemotherapy in an in situ-formed thermal-responsive nanoparticle (TNP) that can co-encapsulate ZnPC and DOX, namely TNP/DOX/ZnPC [46]. The certain synergistic effect of DOX and ZnPC in hydrogel was demonstrated both in vitro at cellular level and in vivo on a 5637 cells xenograft mouse model, indicating that PDT combined with chemotherapeutic drugs is a promising combination therapy for BC.

**Fig. 4:**
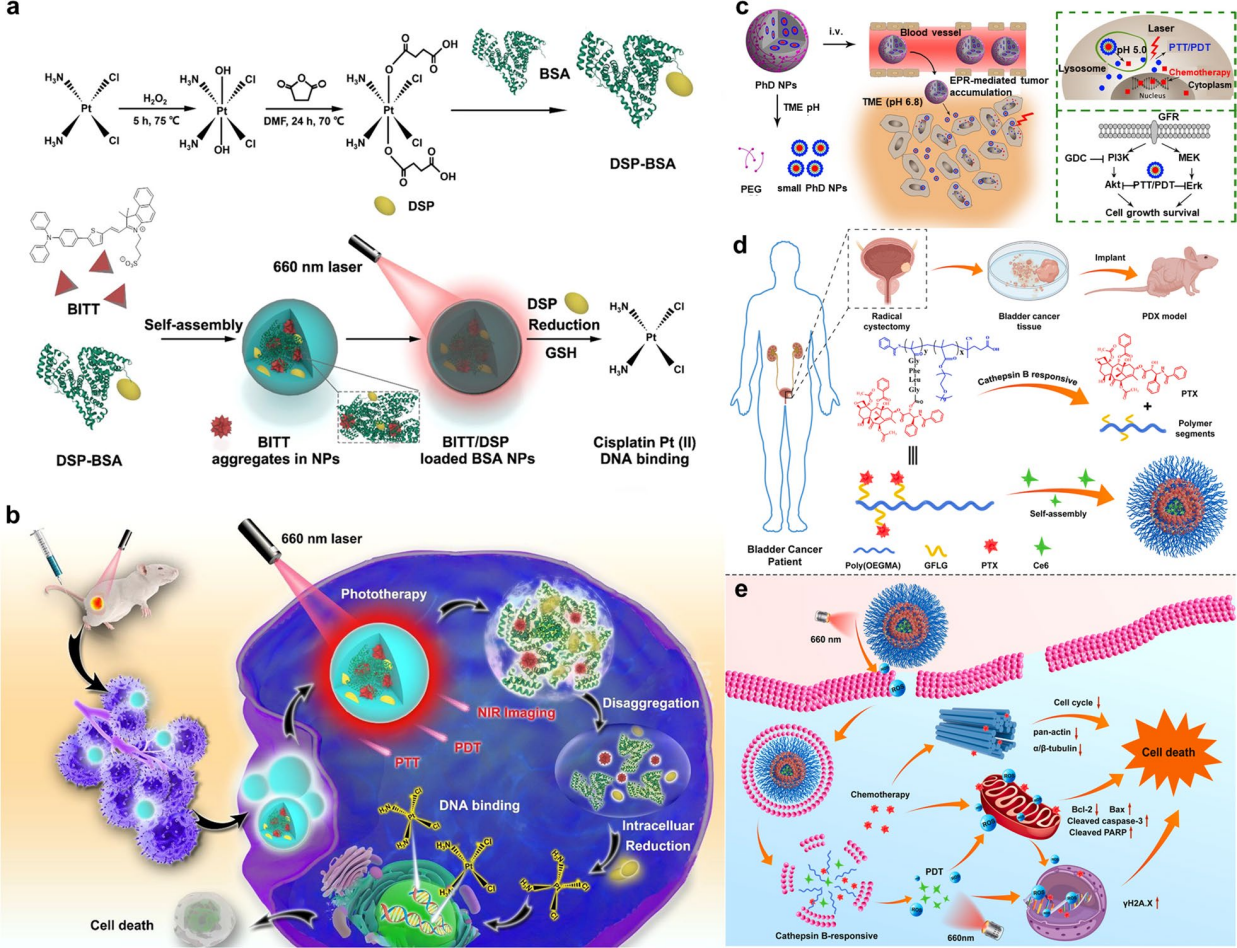
**a** Schematic illustration of the synthesis of BITT@BSA-DSP NPs and cisplatin release. **b** Strategy used for NIRFI-guided photo-enhanced chemotherapy for bladder cancer. Reprinted with permission [47]. Copyright 2022, American Chemical Society. **c** Schematic illustration of two-stage degradation of PhD NPs to release DOX and PhD NPs-mediated photochemotherapy blocked both Akt and Erk pathways to overcome the resistance of chemotherapy and PI3K inhibitor (GDC-0941). Reprinted with permission [48]. Copyright 2020, John Wiley and Sons. **d** Schematic illustration of the construction of a bladder cancer PDX model, degradation of the poly (OEGMA)-based polymer prodrug in response to cathepsin B to release PTX and self-assembly of NPs@Ce6. **e** NPs@Ce6-mediated PCI and PDT under short- and long-term irradiation, respectively, have a synergistic anti-tumor effect with PTX-mediated chemotherapy and induce cell death by blocking cell mitosis, promoting cell apoptosis and damaging DNA. Reprinted with permission [49]. Copyright 2021, Elsevier

Ding et al. combined cisplatin, which is widely used in BC, with PDT and PTT through self-assembled NPs [47]. Aggregation-induced emission luminogens (AIEgens) have been widely studied as PSs for tumor treatment [98]. BITT is a kind of AIEgens, which could not only overcome the aggregation-induced quenching effect of traditional PSs, but also provide the function of near-infrared fluorescence imaging (NIRFI). To reduce the side effect of cisplatin, they employed Pt-2COOH (DSP), a cisplatin(IV) prodrug that can be reduced to cisplatin (II) by a large amount of reducing agents such as GSH in cancer cells [99]. Biocompatible and biodegradable bovine serum albumin (BSA) was applied as a nanocarrier, loaded with BITT and DSP, to construct a NIRFI-guided photo-enhanced BC treatment (Fig. 4a, b). The in vitro and in vivo experimental results demonstrated that the multifunctional BITT@BSA-DSP NPs can promote efficient visualization of tumor regions and significantly inhibit bladder tumor growth.

In terms of overcoming drug resistance, a novel multifunctional nanoparticle, Pheophorbide a (Ppa)-hydrazone-DOX (PhD) NPs, was developed by self-assembly of dimers formed by DOX conjugated to Ppa through a hydrazone bond, with PEGylation cross-linkage introduced onto the surface of NPs [48]. The PhD NPs exhibited a pH-responsive feature because the hydrazone bond is relatively stable under physiological pH conditions and can be cleaved to release DOX in an acidic (pH 5.0–6.0) TME (Fig. 4c). Furthermore, Ppa could mediate PDT and PTT upon laser activation to overcome the drug resistance. Based on a previous research, the BL269 xenografts BC model with a PI3K mutation that was initially sensitive to a PI3K inhibitor (GDC-0941) later developed resistance to the PI3K inhibitor due to the upregulation of extracellular signal-regulated kinase (Erk) pathway [94]. Herein, it was confirmed in chemo- and GDC-resistant PDX BC models that PhD NPs-mediated-photochemotherapy suppressed both Erk and protein kinase B (Akt) pathways to overcome drug resistance and resulted in BC eradication (Fig. 4c) [48].

Moreover, in order to facilitate the effect of drug on the tumor, Tan et al. designed an enzyme-responsive multifunctional nanoparticle, poly (OEGMA)-PTX prodrug@Ce6 (NPs@Ce6), composed of a photosensitizer chlorin e6 (Ce6) and a cathepsin B-responsive polymer-paclitaxel (PTX) prodrug by self-assembly (Fig. 4d) [49]. The polymer prodrug poly (OEGMA)-PTX can be cleaved to specifically release PTX by cathepsin B that is relatively overexpressed in BC cells. Ce6 could mediate both photochemical internalization (PCI) induced by short-term irradiation and PDT induced by long-term irradiation. The PCI effect contributed to cellular uptake and tissue penetration of NPs@Ce6, and thus the chemo-PDT enhanced by two-stage irradiation showed synergistic tumor inhibition in PDX BC models and induced cell death by blocking cell mitosis, promoting cell apoptosis and damaging DNA (Fig. 4e).

###### Combination with targeted therapy

**Fig. 5:**
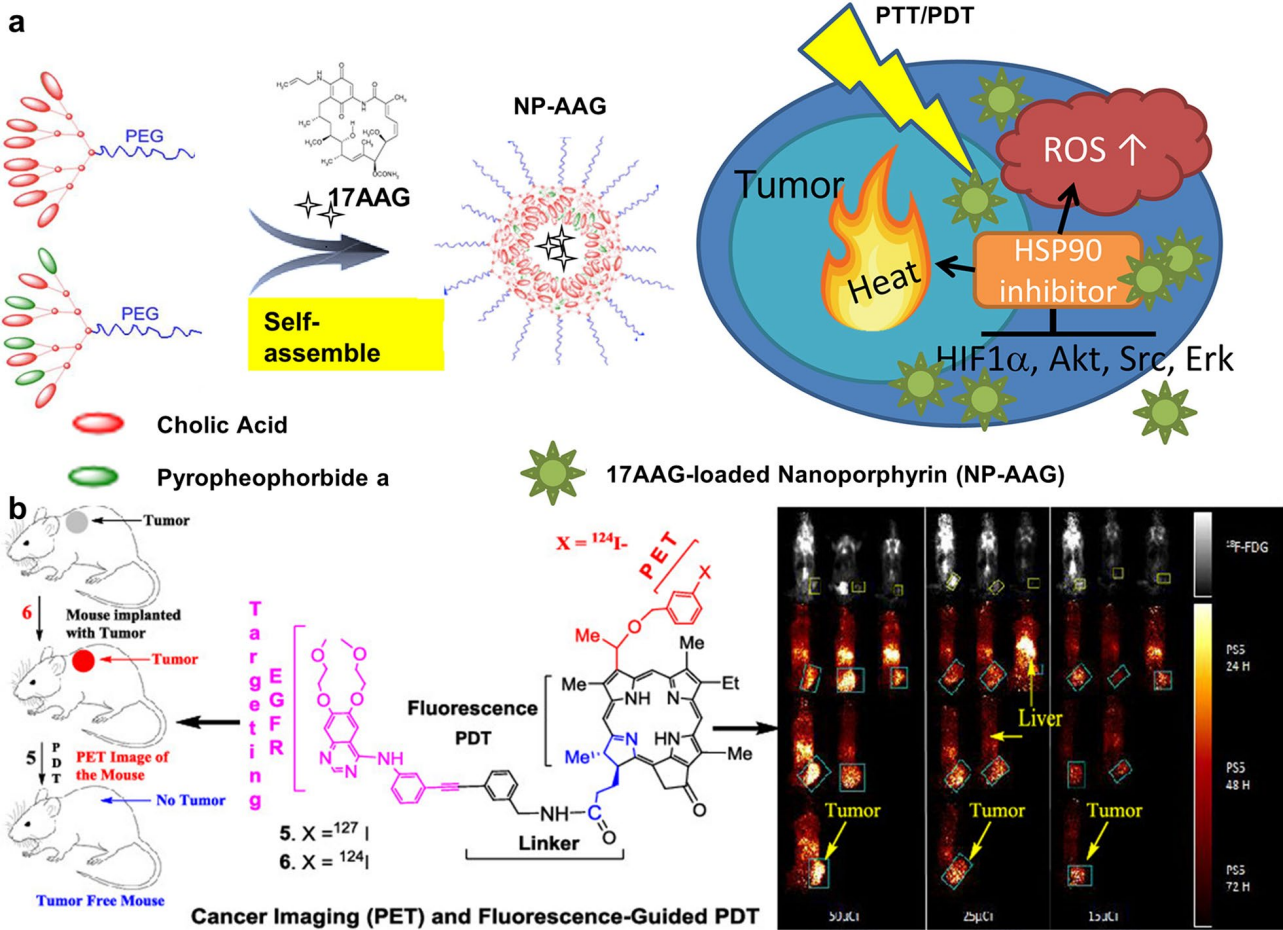
**a** Schematic illustration of self-assembly of nanoporphyrin loaded with 17-AAG and it-mediated trimodal therapy (PDT, PTT and targeted therapy) blocked HIF-1α, Akt, Src and Erk pathways. Reprinted with permission [50]. Copyright 2018, Elsevier. **b** Schematic illustration of the structure of erlotinib-photosensitizer conjugates and conjugates-mediated PET imaging and fluorescence-guided PDT for tumor eradication. Reprinted with permission [52]. Copyright 2019, American Chemical Society

Heat shock protein 90 (HSP90) functions as molecular chaperones, guiding the folding, intracellular disposition, and proteolytic turnover of many key regulators of cell growth, differentiation, and survival. HSP90 is constitutively overexpressed in tumor cells and is considered critical for tumor cell growth and survival, which has been evaluated as an important target for cancer therapy [100, 101]. Studies have shown that the combination of an HSP90 inhibitor 17-allylamino-17-demethoxygeldanamycin (17-AAG) with PDT can improve the antitumor effect of PDT, because PDT can induce the expression of various pro-survival and angiogenic signaling pathway proteins, such as Akt, hypoxia-inducible factor 1α (HIF-1α) and vascular endothelial growth factor (VEGF) in tumor tissues, which are HSP90-dependent client protein [101–103]. Li and his coworkers designed a novel 17-AAG-loaded nanoporphyrin platform (NP-AAG), improving the treatment outcome of PCa by integrating PDT, PTT and targeted therapy in a nanoparticle [103]. Inspired by its efficacy in PCa, they continued to investigate the synergistic effect of NP-AAG against BC cell lines, and further demonstrated its excellent efficacy on a PDX BC mouse model [50]. The improved efficacy is attributed to the targeted delivery of 17-AAG to tumor tissues, which not only inhibited the PDT/PTT-induced HIF-1α, but also down-regulated several cancer-promoting signaling molecules, such as Akt, Src and Erk in BC (Fig. 5a).

One of the potential therapeutic targets of BC is epidermal growth factor receptor (EGFR), which is up to 74% overexpressed in BC tissue specimens but lower in normal urothelium [104, 105]. Overexpression of EGFR in BC has been shown to be associated with poor clinical prognosis [106]. EGFR is located in the basal layer of urothelial cells in normal urothelium, but is present in both the luminal and basal layers of urothelial cells in BC [107], making targeted intravesical therapy a potential option for BC treatment. Two main types of EGFR inhibitors have been reported, including monoclonal antibodies [108], and tyrosine kinase inhibitors [109]. Railkar et al. reported a molecular targeted PDT named photoimmunotherapy (PIT) targeting EGFR-expressing BC cells via conjugating the anti-EGFR humanized antibody panitumumab with a PS, IRDye700Dx (IR700) [51]. IR700 is different from traditional PSs and itself has no therapeutic effect for its hydrophilic property preventing it from freely entering cells, thereby avoiding generalized phototoxicity when activated by NIR light. At the same time, panitumumab-IR700 conjugates can selectively bind to EGFR-expressing cells and induce cell death upon NIR activation. They demonstrated the panitumumab-IR700-induced PIT leads to cell death in EGFR-expressing cells and the cytotoxic effect depends on the amount of EGFR expressing in cells. Cheruku et al. fabricated an EGFR-targeted multifunctional PS, showing EGFR target specificity by being conjugated with an erlotinib moiety [52]. Erlotinib is a tyrosine kinase inhibitor that has been clinically approved. The erlotinib-PS conjugates showed excellent long-term tumor cure in combination with positron emission tomography (PET) imaging ability in severe combined immunodeficient (SCID) mice bearing UM-UC-3 (EGFR-positive) tumors (Fig. 5b).

It has been reported that BC is one of the most molecularly heterogeneous cancers with a high mutation rate [110]. Therefore, PIT with combined targets may have a stronger therapeutic effect on BC than PIT with a single target. HER2 is another potential target for PIT and is mainly overexpressed in the luminal subtypes of BC, while the basal/squamous subtypes of BC show enrichment in EGFR [111]. A combination of EGFR- and HER2-targeted PIT was proposed to target a broader range of bladder tumors with IR700-conjugated panitumumab (pan) and trastuzumab (tra), respectively [53]. Combination of pan-IR700 and tra-IR700 showed the strongest antitumor effects than either agent alone both in vitro and in vivo.

Expression of EGFR was only detected in BC with squamous differentiation and pure squamous cell carcinomas of bladder [112, 113], while CD47 was found expressed in all human bladder tumors examined in characterization of bladder tumor-initiating cells [114], with absence from luminal normal urothelium [115], making it a more broadly applicable BC target for PIT. CD47 is a “don’t eat me” marker on the surface of all human solid tumor cells and functions as a ligand for signal regulatory protein-α (SIRPα), a protein expressed on macrophages and dendritic cells [116, 117]. Monoclonal antibodies (mAbs) that block the interaction between CD47 and SIRPα enabled the phagocytosis of human solid tumor cells in vitro and inhibited both growth and metastasis of tumors in vivo [118]. Anti-CD47-IR700, a mouse anti-human CD47 mAb-IR700 conjugate, mediated CD47-targeted NIR-PIT, that induced light-dose-dependent cytotoxicity in CD47-expressing human BC cell lines and primary BC cells from fresh surgical specimens, and showed prominent in vivo tumor inhibition and resulted in significantly longer survival compared with the control animals after five rounds of PIT [54].

#### PDT for prostate cancer

PCa was estimated to account for almost 1.4 million new cases and 375,000 deaths worldwide, and has become the second most common cancer and the fifth leading cause of cancer death among men in 2020 [21]. Prostate is a small, peanut-like encapsulated organ that helps achieve high local drug concentration and prevent unwanted side effects and toxicity from the extracapsular tissues [119]. It can be accessed by relatively non-invasive procedures, such as transurethral route, transrectal route and transperineal route [120]. The blood perfusion rate to the prostate is relatively slow (i.e., 16 mL per minute per 100 g) [121], compared with other major organs like liver and kidney [122]. These anatomical and physiological features of the prostate make it a potential candidate for localized PDT utilizing nanobiotechnology. Emerging strategies of nanobiotechnology-enhanced PDT for PCa over the past 10 years are summarized in Table 2.

**Table 2:** Emerging strategies of enhanced PDT for prostate cancer

Photosensitizers	Biological model	Strategy	Result	Refs.
Anti-PSMA-IR700	Subcutaneous PC-3 in nude mice	Anti-PSMA monoclonal antibody conjugation	Inhibited tumor growth and prolonged survival significantly	[123]
^111^In-DTPA-D2B-IRDye700DX	Subcutaneous PSMA^+^ LS174T-PSMA cells or PSMA^−^ LS174T-wildtype cells in nude mice	Anti-PSMA monoclonal antibody conjugation; being radiolabeled with ^111^In	Fluorescence and radioactivity dual tumor imaging capabilities, and tumor suppression	[124]
PSMA-1-Pc413, PSMA-1-IR700	Subcutaneous PC-3PIP in nude mice	A novel PSMA-targeted peptide conjugation	Fluorescence imaging and tumor elimination capability	[125]
[^111^In]In-DOTA(GA)-IRDye700DX-PSMA ligands	Subcutaneous LS174T-PSMA in the left flank and LS174T in the right flank in nude mice	Variants of PSMA-targeted ligands conjugation; being radiolabeled with ^111^In	Fluorescence and radioactivity dual tumor imaging capabilities;	[126]
PSMA-617, PSMA-1007	Subcutaneous PSMA^+^ LS174T-PSMA cells or PSMA^−^ LS174T-wildtype cells in nude mice	Three new PSMA-targeted ligands conjugation; being radiolabeled with ^111^In	Enhanced tumor targeting and enabled multimodal image-guided prostate cancer surgery combined with PDT	[127]
bbp	Subcutaneous PC-3 PIP and PC-3 in nude mice	A PSMA-targeted ligand conjugation; a peptide linker modification to prolong plasma circulation time	Enhanced tumor-inhibition rate with NIR fluorescence image guidance	[128]
YC-9	Subcutaneous PC-3 PIP in nude mice	A PSMA-targeted peptide conjugation	Delayed tumor growth in PC-3PIP tumor mice	[129]
RGD-L-Pyro 1, RGD-L-Glu-Pyro 2	Subcutaneous PC-3 in nude mice	An integrin-binding sequence (cRGD) conjugation; added hydrophilic PEG and hydrophilic carboxylic acid group as the linker to enhance the water solubility of the conjugate	PDT-mediated PC-3 tumors eradication	[130]
PpIX-PA	Subcutaneous PC-3 in nude mice	PAs conjugation to increase accumulation in tumor cells and ROS quantum yield	More cancer cells apoptosis than PpIX alone	[131]
uPA-PPP	Subcutaneous PC-3 in Swiss Nu/Nu mice	uPA-responsive prodrug	ROS-mediated tumor cell eradication with bioluminescence imaging guide	[77, 78]
PGL-MBs	Subcutaneous PC-3 in nude mice	Combination with microbubbles to realize US imaging and promote the delivery of PGL under LFUS	Excellent therapeutic efficacy with US imaging guide	[76]
PTX-PP@Au NPs	Subcutaneous PC-3 in nude mice	Combination with PTT, chemotherapy and ion channel inhibition	Achieving the therapy to ARPC with low toxicity on liver function or other organs	[132]
GNS@IR820/DTX-CD133	Subcutaneous PC-3 in nude mice	The CD133 antibody conjugation; Combination with PTT, chemotherapy, NIRFI and PAI	Achieving the excellent antitumor effects of the synergistic PTT/PDT/ chemotherapy strategies under the NIR-light irradiation	[133]
HSA@IR780@DTX	Subcutaneous 22RV1 in nude mice	Combination with PTT, and chemotherapy	Complete xenografted prostate tumor inhibition after NIR-light irradiation	[134]
17-AAG-loaded nanoporphyrin	Subcutaneous PC-3 in nude mice	Combination with NIRFI, PTT and HSP90-targeted therapy	Light-dose dependent tumor inhibition	[103]

*PSMA* Prostate-specific membrane antigen; *IR700* IRDye700Dx; *DTPA* Diethylenetriaminepentaacetic acid; *PC-3PIP* PSMA^+^PC-3 cells; *PDT* Photodynamic therapy; *BBP* BChI-Based PSMA-targeted photosensitizer; *NIR* Near infrared; *PEG* Polyethylene glycol; *PpIX* Protoporphyrin IX; *PA* Polyamine; *ROS* Reactive oxygen species; *uPA* Urokinase plasminogen activation; *PGL* Porphyrin-grafted lipid; *MBs* Microbubbles; *US* Ultrasound; LFUS: Low-frequency ultrasound; *PTX* Paclitaxel; *PP* Pluronic-polyethylenimine; *Au* Aurum; *PTT* Photothermal therapy; *ARPC* Androgen-resistant prostate cancer; *GNS* Gold nanostars; *DTX* Docetaxel; *NIRFI* Near-infrared fluorescence imaging; *PAI* Photoacoustic imaging; *HSA* Human serum albumin; *HSP90* Heat shock protein 90

##### Improvement of targeted tumor cellular internalization

###### Cell surface protein-targeted delivery

**Fig. 6:**
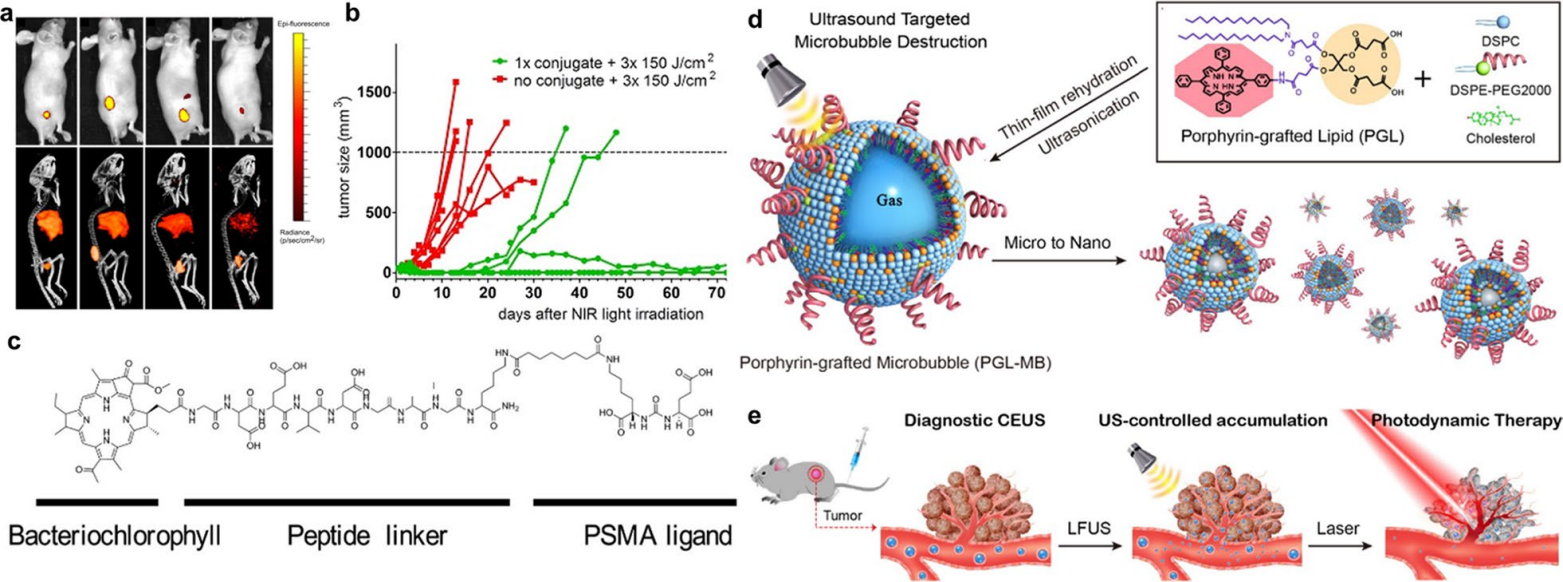
**a** NIRF (top) and µSPECT/CT (bottom) in mice (different mice per time point were used) with s.c. LS174T-PSMA tumors at 24, 48, 72, and 168 h after injection of 30 µg of ^111^In-DTPA-D2B-IRDye700DX. **b** Comparison of tumor growth in mice irradiated with the highest NIR light dose (3 × 150 J/cm^2^) only (control group) and mice treated with a single administration of the conjugate followed by NIR light exposure of 3 × 150 J/cm^2^. Reprinted with permission [124]. Distributed under a Creative Commons Attribution (CC BY-NC 4.0) license. **c** Chemical structure of BChl-peptide-PSMA (BPP). Reprinted with permission [128]. Copyright 2019, John Wiley and Sons. **d** Schematic illustration of the preparation of PGL-MB and its transformation from microbubbles to nanoparticles under exposure to low-frequency ultrasound (LFUS). **e** Schematic illustration of in vivo PDT under the guidance of contrast enhance ultrasound (CEUS) imaging, followed by US-controlled accumulation. Reprinted with permission [76]. Distributed under a Creative Commons Attribution (CC BY-NC 4.0) license

Prostate-specific membrane antigen (PSMA), overexpressed on the membrane surface of most PCa cells instead of normal tissue cells, is a sort of type II transmembrane glycoprotein receptor possessing sequence and structural homology with transferrin receptors [135]. Given the specificity of its distribution, numerous ligands or antibodies have been developed to be applied to various cancer imaging and treatment methods for increasing targeting and efficacy [136]. Tadanobu et al. synthesized IR700-conjugated anti-PSMA as a novel mAb-photo-absorber conjugate (APC) by combining hydrophilic silica-phthalocyanine dye-IR700 with anti-human PSMA [123]. The innovative APC demonstrates considerable tumor cell killing and fluorescence imaging in vitro and vivo models. Lutje et al. conjugated the anti-PSMA mAb D2B labeled with ^111^In with the photosensitizer IRDye700DX (called ^111^In-DTPA-D2B-IRDye700DX) for both pre- and intra-operative tumor localization and eradication of (residual) tumor tissue [124]. The dual imaging capability of radionuclide and NIR fluorescence imaging and photodynamic efficacy were validated in the mouse xenograft model (Fig. 6a, b).

Movement of small-Molecule PSMA inhibitors is another therapeutic strategy to target PSMA. Compared with antibodies, small-molecule PSMA ligands hold advantages of easy synthesis and low cost. Most structures of small-molecule PSMA ligands are based on glutamate–urea–lysine dimers and phosphonamidothionate derivatives of glutamic acid [137]. Several researches combine photosensitizers with PSMA-specific ligands to enhance the distribution of photosensitizers at tumor sites. Wang et al. formed two sorts of PSMA-targeting PDT conjugates named PSMA-1-Pc413 and PSMA-1-IR700 by connecting Pc413 (a kind of phthalocyanine analogs) and IR700 with a peptide-based negatively charged PSMA ligand (PSMA-1, which the amino acid sequence is Glu-CO-Glu'-Amc-Ahx-Glu-Glu-Glu-Lys-NH_2_) [125]. Derks et al. synthesized 13 novel multimodal glutamate-urea-lysine-base PSMA-targeting ligands using solid-phase chemistry, and the ligands were coupled to IRDye700DX and labeled with ^111^In [126]. They demonstrated that the best ligands—N064, N140 and N142 showed excellent radionuclide and fluorescence imaging performance in PSMA-positive tumors and great photodynamic efficacy. In their another study, the glutamic acid of PSMA-617, PSMA-1007 (the current PSMA targeting tracers) was replaced with a lysine residue for enhancing molecular tumor targeting [127]. Linker modification significantly improved tumor accumulation compared to previously developed PSMA-N064 ligand. In view of the high extinction coefficients in the NIR region, bacteriochlorophyll (BChI) derivatives are more applicable for imaging and photodynamic therapy than many photosensitizers for tumors located at a distance from the skin [138]. Overchuk et al. developed a BChI-based PSMA-targeted photosensitizer (BPP) [128]. BBP was consisting of three building blocks: (1) a PSMA-affinity ligand, (2) a peptide linker to prolong plasma circulation time, and (3) a BChI photosensitizer (Qy absorption maximum at 750 nm) (Fig. 6c). The 9 D-peptide linker prolonged the plasma circulation time (12.65 h) of BPP and increased the accumulation of photosensitizers at tumor sites. In a subcutaneous prostate adenocarcinoma mouse model, BPP demonstrated precise image-guided photodynamic treatment. Chen et al. synthesized a low-molecular-weight theranostic photosensitizer called YC-9 by conjugating IRDye700DX N-hydroxysuccinimide (NHS) ester with a PSMA targeting Lys-Glu urea through a lysine-suberate linker in suitable yield [129]. Significant tumor growth delay and extended median survival of the PSMA^+^ PC-3-PIP tumor mice were observed by PDT with YC-9.

Integrins are heterodimeric proteins with α and β chains, anchored on the cell surface and involved in intercellular adhesion and signal transduction. Numerous studies have shown that integrin α_V_β_3_ is overexpressed in solid tumors and on neovascular endothelium and is activated by thyroid hormones to activate downstream Erk1/2 [139, 140]. The integrin α_V_β_3_ signaling network promotes the proliferation and metastasis of various tumor cells [21, 141]. In light of the differential expression of integrin α_V_β_3_ between tumor and normal tissue, Li et al. combined pyropheophorbide-a (Pyro) with a cyclic cRGDfk (cRGD) peptide, an integrin-binding sequence, to improve the targeting ability of Pyro [130]. A highly hydrophilic PEG chain and an extra strongly hydrophilic carboxylic acid group were used as the linker to optimize the pharmacokinetics of the compound in blood. A magnificent tumor enrichment property and tumors tumor elimination after only one dose of PDT have been observed in the xenograft murine tumor model.

ALA-based photodynamic technology has been proven to have broad clinical value in PDT and PD. Intracytoplasmic ATP-binding cassette (ABC) subfamily B member 6 (ABCB6) transports exogenous ALA to mitochondria to produce PpIX. In tumor cells, activation of the ALA influx transporter of peptide transporter 1 (PEPT1) and inactivation of ABC superfamily G member 2 (ABCG2), which excretes PpIX, further exacerbates the accumulation of PpIX in tumor cells. PpIX is photoactive and produces red fluorescence in tumor cells [142]. Fidanzi-Dugas et al. conjugated PpIX with polyamines (PAs) to synthesize a novel photosensitizer PpIX-PA because PAs were actively transported into cancer cells through the up-regulated polyamine transport system (PTS) expressed on the cell surface [131]. The experimental results showed that PpIX-PA could induce the intrinsic pathway of apoptosis in vitro and inhibit tumor growth in a nude mouse xenograft model.

###### Endogenous stimulus-responsiveness

As a member of the serine protease family, the urokinase plasminogen activation (uPA) has been confirmed by several researchers to play a role in promoting the invasion of PCa [143–145]. uPA is activated by binding to uPA receptors (uPAR) and converts the inactive enzyme plasminogen into the active serine protease plasmin, which engages in the degradation of extracellular matrix (ECM) and promotes cancer invasion and metastasis [146]. A high abundance of uPA in PCa site can act as an activator of photosensitizer prodrugs. Zuluaga et al. synthesized innovative uPA-responsive photosensitizer prodrugs named uPA-PPP by conjugating pheophorbide *a* with peptide linkers that can be cleaved by uPA [77]. Different side chain modifications (mPEG 20 kDa, mPEO_4_ and mPEO_8_) were used to improve the water-solubility and bioavailability of uPA-PPP. In their another research, they evaluated the in vitro phototoxicity of uPA-PPP in PC-3 cells and luciferase-transfected PC-3 M-luc-C6 cells and in a PCa xenograft model [78]. Prodrugs alone (8 μmol/L) had no effect on the viability of PC-3 cells. In vivo experiments showed that the systemic use of the prodrugs resulted in a strong fluorescent signal at the tumor site, indicating the localization and selective activation of the prodrugs in tumor site. High Performance Liquid Chromatography (HPLC) analysis of tissue extracts verified that the most photoactive Pba-GSGR fragment after enzymatic cleavage was present in the tumor site and the content in skin or muscle is very low.

###### Exogenous stimulus-responsiveness

Conventional ultrasound imaging is difficult to accurately identify PCa lesions or detect and evaluate treatment effects [147]. Due to the presence of the gas core in the structure of microbubbles (MBs), they are widely used as contrast agents for ultrasound imaging to improve the visualization of tumor lesions [148]. Second, the use of low-frequency ultrasound (LFUS) to induce cavitation in MBs can facilitate drug delivery by forming transient pores on the cell surface [149]. In view of the above advantages, You et al. developed a new strategy to combine MBs with PDT—using porphyrin-grafted lipid (PGL) mixed with inert fluorocarbon gas to fabricate MBs (PGL-MBs) (Fig. 6d) [76]. Under LFUS, PGL-MBs showed excellent contrast enhancement for US imaging and were converted into PGL-NPs, avoiding the quenching of porphyrin fluorescence. In addition, the ultrasound-induced sonoporation effect made PGL-NPs further accumulate in the tumor tissue to optimize the PDT effect, which resulted in significant tumor suppression in vitro and in vivo (Fig. 6e).

##### Combination therapy

###### Combination with chemotherapy

**Fig. 7:**
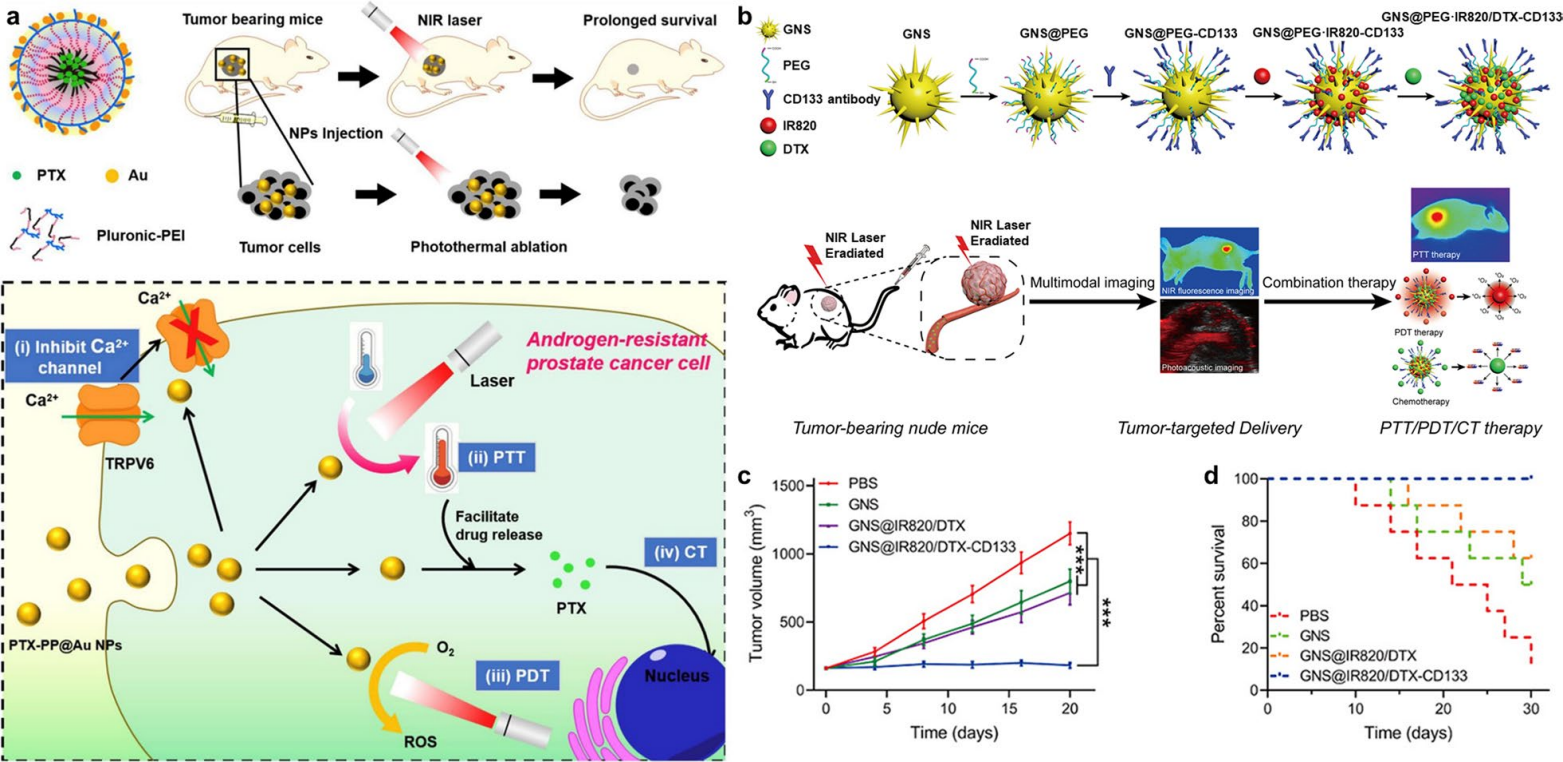
**a** Schematic illustration of gold-caged copolymer nanoparticles as the synergistic PTT/PDT/chemotherapy platform and their potential therapeutic mechanism against androgen-resistant prostate cancer cells. Reprinted with permission [132]. Copyright 2019, Elsevier. **b** Schematic illustration of the preparation of GNS@IR820/DTX-CD133 and its synergistic combination therapy (PTT/PDT/chemotherapy) for CRPC under the monitoring of multimodal imaging. **c** The tumor volume in various groups with NIR-light irradiation. **d** Survival curves of tumor-bearing nude mice in various groups after NIR-light irradiation treatment. Reprinted with permission [133]. Copyright 2020, Elsevier

Gold nanoparticles (GNs) are widely used in cancer diagnosis and treatment due to their superior physical properties [150]. In brief, GNs have the advantages of easy surface modification, controllable particle size, high drug loading, high biocompatibility and so on, which endows GNs with an upsurge of researches in the fields of photothermal, photodynamic, chemotherapy and immunotherapy for solid tumors [151, 152]. Wang et al. encapsulated the chemotherapeutic agent paclitaxel (PTX) in copolymer Pluronic-polyethylenimine (Pluronic-PEI) and the system was covered by a gold cage (Fig. 7a) [132]. The synthesized PTX-PP@Au NPs possessed 4 functions to realize the combination of PTT/PDT/chemotherapy: 1) the Pluoronic-PEI assembling into micelles was used as an encapsulator for drugs and provided reduction sites for the gold cage to weaken toxicity; 2) gold cages with surface plasmon resonance peak at NIR region in a broad window qualifying the PTT/PDT potentiality; 3) chemotherapy of PTX; 4) the expression of transient receptor potential cation channel subfamily V member 6 (TRPV6) was inhibited for revising androgen resistance. Moreover, Tan et al. loaded IR820 and docetaxel (DTX, a specific cell cycle chemotherapeutic agent) on gold nanostars (GNS) coated with PEG and functionalized with CD133 antibody to obtain GNS@IR820/DTX-CD133 (Fig. 7b) [133]. CD133 is a membrane glycoprotein expressed on the surface of PCa stem cells (PCSCs), which have been linked to the occurrence of castration resistant prostate cancer (CRPC) [153]. The PEG modified on the nanoplatform enhanced its drug loading efficiency and acted as the attachment site of CD133 antibody for improving the tumor site targeting ability. The synthetic nanoplatform integrating the PTT/PDT/chemotherapy strategies with NIR fluorescence and photoacoustic imaging (PAI) achieves excellent antitumor effects of CRPC (Fig. 7c, d).

Lian et al. encapsulated IR780 iodide and DTX in HSA to form HSA@IR780@DTX for combining PTT and PDT with chemotherapy and fluorescence imaging [134]. The hydrophobic drug DTX and IR780 induced the self-assembly of HSA through the hydrophobic interaction with the hydrophobic domain of HSA. The xenografted prostate tumors on mice treated with HSA@IR780@DTX under NIR laser irradiation were eliminated completely compared to those treated with chemotherapy alone (HSA@DTX and HSA@IR780@DTX without laser) or PTT/PDT alone (HSA@IR780 with laser).

#### PDT for kidney cancer

##### Combination therapy

There were an estimated 431,000 new cases and 179,000 deaths of kidney cancer worldwide in 2020 [21], and renal cell carcinoma (RCC), a malignant tumor originating from the tubular epithelium of the renal parenchyma, accounted for more than 90% of such malignancies [154]. As the most lethal of the common urological cancers, RCC is mainly treated by surgery because overall response rates and durable complete responses to immunotherapy and targeted therapy remain rare for this chemoresistant cancer [155]. There is a large demand for new treatments for RCC. A facilely prepared core–shell structured TiO_2_@red phosphorus nanorods (TiO_2_@RP NRs) that can effectively mediate the combined PDT and PTT for clear cell RCC (ccRCC) was reported [93]. Red phosphorus (RP), a new class of biophotocatalysts, displaying great absorption of NIR [156], was deposited onto the TiO_2_ NR surface to extend the absorption range of TiO_2_@RP NRs to NIR. Owing to the synergistic effect of PDT and PTT, TiO_2_@RP NRs could kill both in vitro ccRCC cells and in vivo deep ccRCC tumors.

### SDT for urological cancers

Unlike PDT, the therapeutic effect of SDT relies on the low intensity focused ultrasound-mediated sonosensitizer activation, which further catalyzes the generation of ROS [157]. At present, the exact mechanism of ultrasonic-mediated ROS generation has not been determined yet. There are several possible mechanisms such as sonoluminescence, pyrolysis, cavitation, and ROS-independent cytotoxicity [158]. Although many mysteries remain to be solved, SDT still attracts the attention of researchers as it can subtly circumvent two thorny issues that hinder the clinical application of PDT: 1) the weak tissue penetration of light, 2) phototoxic side effects when photosensitizers trapped in the skin are exposed to sunlight. In addition, in the treatment of urological tumors (such as PCa), unlike PDT requiring the insertion of an optical fiber from the urethra [159], SDT only needs to place the ultrasound probe on the body surface, which will undoubtedly reduce the pain of the patient during treatment and enhance the treatment adherence. However, the quantum yield of ROS in SDT is relatively lower than PDT, which means there is still much room for the development of sonosensitizers.

Various types of sonosensitizers, including organic molecules, inorganic nanomaterials, hybrid materials and metal-based particles, have been extensively investigated in the past few decades, but finding or synthesizing sonosensitizers with high ROS conversion efficiency is still a research hotspot [18, 160]. In addition to using SDT alone, researchers also hope to achieve a synergistic treatment effect on tumors by combining other therapies, such as PDT, PTT, and chemotherapy [80, 161, 162]. Unfortunately, there is not a consensus on the standardization of ultrasound parameters and experimental setups for performing SDT in vitro or in vivo so far [163]. Emerging strategies of nanobiotechnology-enhanced SDT for BC and PCa over the past 10 years are summarized in Table 3.

**Table 3:** Emerging strategies of enhanced SDT for BC and PCa

Sonosensitizers	Biological model	Strategy	Result	Refs.
AMVs	Subcutaneous patient-derived bladder tumor tissue sample in nude mice	Combination of AIE-active sonosensitizer and patient-derived MVs	The superior tumor targeting ability and efficient personalized SDT therapy on PDX models	[81]
CAT-TCPP/FCS NPs	Orthotopic MB49 in C57BL/6 mice	CAT loading to alleviate tumor hypoxia; FCS as an effective transmucosal delivery carrier	Orthotopic bladder tumors under US without systemic toxicity	[71]
MVSN-IR825	Subcutaneous and orthotopic PC-3 in nude mice	Combination of virus‐mimic surface topology and MB‐assisted LFUS; Combination of PAI, FI, and MRI; combination with PDT and PTT	A successful combined anticancer effect with trimodal imaging to determine the optimal therapeutic timing	[82]
CS-ss-IR806 (CSR)	Subcutaneous PC-3 in nude mice	Redox- and hyaluronidase-responsive drug delivery; combination with PDT and PTT	Superior trimodal anticancer efficacy after dual-irradiation compared with either monoirradiation strategy	[80]
HPNPs	Subcutaneous LNCaP in nude mice	pH- and cathepsin B- responsive drug delivery	SDT-mediated tumor elimination with no adverse effects	[79]
HHSN-C/P-mAb	Subcutaneous PC-3 in nude mice	PSCA monoclonal antibody conjugation; pH-responsiveness; combination of US imaging and MRI; combination with chemotherapy	High-effective synergistic therapy	[164]
TiO_2_: Gd@DOX/FA	Subcutaneous LNCaP in nude mice	pH-responsive drug delivery; MRI; combination with chemotherapy	Smaller tumor sizes of all the nanomedicine groups than free dox (v:v_0_ = 4.2)	[162]

*AIE* Aggregation-induced emission; *MVs* Microvesicles; *SDT* Sonodynamic therapy; *PDX* Patient-derived xenograft; *CAT* Catalase; *TCPP* Meso-tetra(4-carboxyphenyl) porphine; *FCS* Fluorinated chitosan; *MB* Microbubble; *LFUS* Low-frequency ultrasound; *PAI* Photoacoustic imaging; *FI* Fluorescence imaging; *MRI* Magnetic resonance imaging; *PDT* Photodynamic therapy; *PTT* Photothermal therapy; *CS* Chondroitin sulfate; *PSCA* Prostate stem cell antigen; *US* Ultrasound; *DOX* Doxycycline; *FA* Folic acid

#### SDT for bladder cancer

##### Improvement of targeted tumor cellular internalization

###### NPs with biomimetic surfaces

**Fig. 8:**
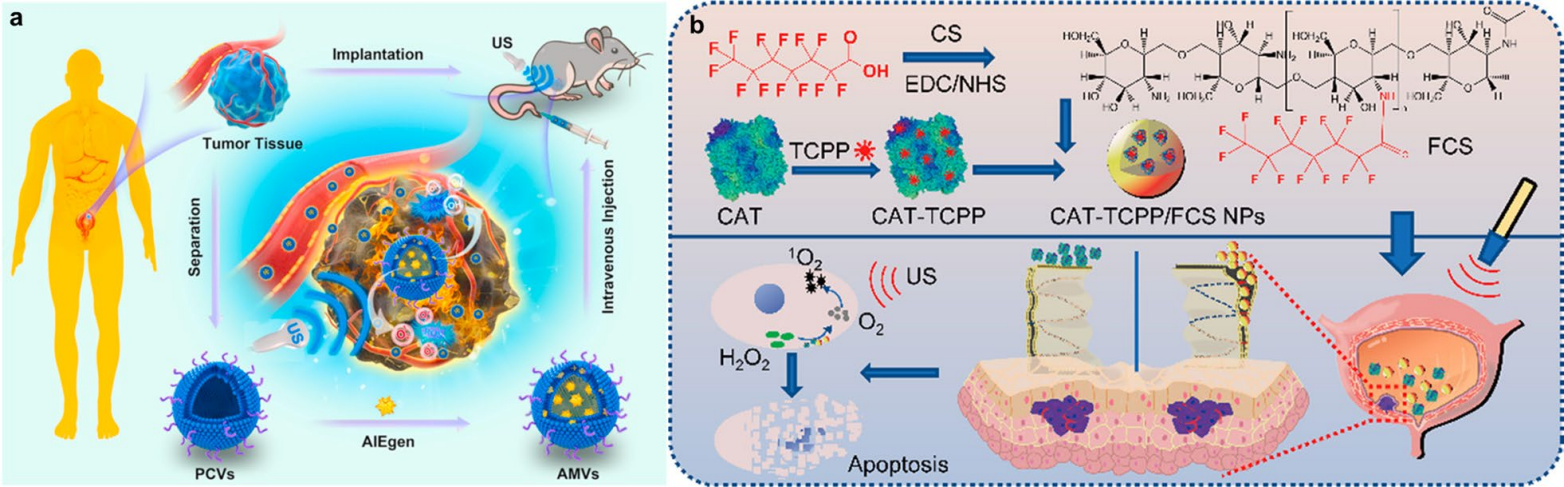
**a** Schematic illustration of patient-derived microvesicles/AIE Luminogen hybrid system for personalized sonodynamic cancer treatment in patient-derived xenograft (PDX) models. Reprinted with permission [81]. Copyright 2021, Elsevier. **b** Schematic illustration of the preparation of CAT-TCPP/FCS NPs and their abilities to enhance transmucosal delivery and improve oxygen generation in SDT for orthotopic bladder tumors. Reprinted with permission [71]. Copyright 2020, American Chemical Society

AIEgens have higher fluorescence quantum yields in aggregated and solid states than in isolated states, which can be designed to exhibit excellent photosensitization, photothermal conversion efficiency and sonodynamic performance [165]. To overcome the hydrophobicity and poor tumor-targeting ability of AIEgens, Duo et al. prepared a patient-derived microvesicles (MVs)/AIEgens hybrid system (AMVs) using DCPy [(E)-4-(2-(7-(diphenylamino)-9-ethyl-9H-carbazol-2-yl) vinyl)-1-methylpyridin-1-ium hexafluorophosphate, AIEgens used in the experiment] as a sonosensitizer in SDT (Fig. 8a) [81]. In vitro experiments show that AMVs have great performance to induce ROS in T24 cells. MVs prepared from patient-derived BC cells enhanced the biocompatibility and tumor-targeting ability of AIEgens, exhibiting tumor regression in PDX models.

##### Hypoxia alleviation

In addition to the fact that TME itself is hypoxic as mentioned above, the process of SDT, like PDT, will continue to consume oxygen and aggravate hypoxia, and generate negative feedback to limit the efficacy of SDT.

###### In situ oxygen generation

Due to the penetrating power of ultrasound, sonodynamic therapy can replace photodynamic therapy that requires the use of an endoscope to enter the bladder. However, the hypoxic microenvironment of the tumor reduces the effect of SDT, which becomes one of the problems to be overcome by sonodynamic therapy [166]. In order to solve this problem, Li et al. developed a transmucosal oxygen-self-production SDT nanoplatform, namely CAT-TCPP/FCS NPs, using fluorinated chitosan (FCS) to encapsulate sonosensitizer meso-tetra(4-carboxyphenyl)porphine (TCPP)-conjugated CAT by self-assembly (Fig. 8b) [71]. The fluorination of the NPs can reversibly modulate the transepithelial electrical resistance (TEER) and open the tight junctions of the bladder epithelium, which improves the penetrating function of NPs in the bladder mucosa and tumor penetration. The loaded CAT enhances in situ O_2_ production and improves the efficiency of SDT to generate ROS and inhibit tumors in orthotopic bladder tumors modal.

#### SDT for prostate cancer

##### Improvement of targeted tumor cellular internalization

###### NPs with biomimetic surfaces

**Fig. 9:**
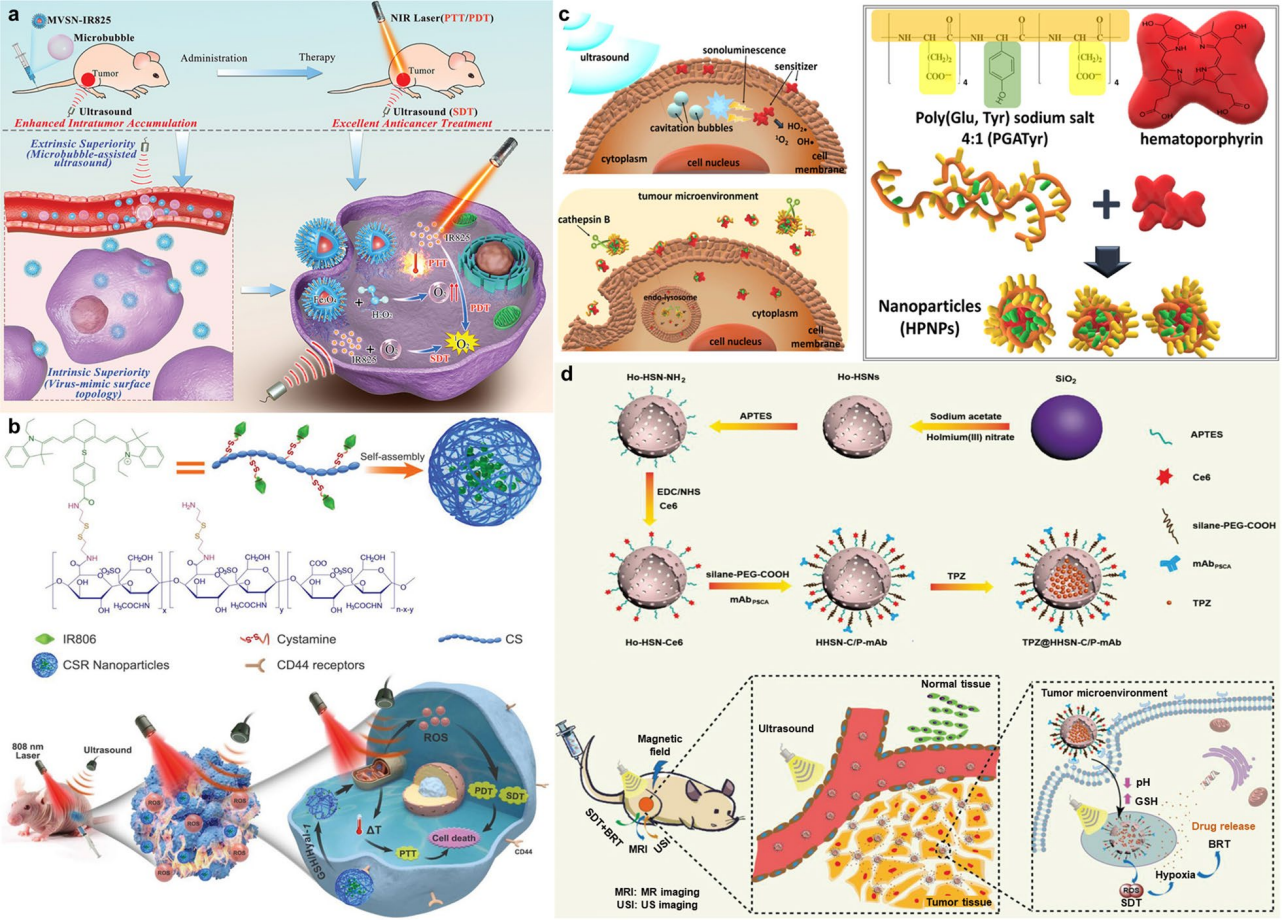
**a** Schematic illustration of the marriage of intrinsic virus-mimic surface topology and extrinsic microbubble‐assisted ultrasound for enhanced intratumor accumulation of MVSN-IR825 and it-mediated anticancer treatment by trimodal PTT/PDT/SDT. Reprinted with permission [82]. Distributed under a Creative Commons Attribution (CC BY 4.0) license. **b** Schematic illustration of construction and self-assembly of CSR NPs and the functions of as-synthesized CSR NPs under dual sono/photoactivation for trimodal SDT/PDT/PTT against localized PCa. Reprinted with permission [80]. Copyright 2021, John Wiley and Sons. **c** Schematic illustration of the suggested mechanism of the cytotoxic effect induced by HPNPs under US irradiation, cathepsin B-responsiveness of HPNPs for improved tumor cellular uptake, and self-assembly of the co-polymer with hematoporphyrin for the formation of HPNPs. Reprinted with permission [79]. Distributed under a Creative Commons Attribution (CC BY 4.0) license. **d** Schematic illustration of the preparation of TPZ@HHSN-C/P-mAb and its application for MRI and US imaging and combined SDT&TPZ for tumors. Reprinted with permission [164]. Copyright 2018, John Wiley and Sons

The development of tumor treatment regimens based on viral structures has attracted extensive attention from researchers. By simulating the surface roughness, charge distribution, and glycosylation modification of the virus structure, the cellular internalization and immune evasion capabilities of the nanoparticles were significantly improved [167]. In view of the advantages above, Wang et al. synthesized virus-like mesoporous silica nanoparticles with a spiky tubular rough surface via a novel single-micelle epitaxial growth approach [168]. The surface topology of the NPs enhanced intracellular uptake capacity. Microbubble‐assisted ultrasound (MAUS) can instantaneously enhance cell membrane permeability and improve intracellular uptake of drugs or nanoparticles utilizing ultrasonic cavitation [169]. Meng et al. developed a novel “intrinsic plus extrinsic superiority” strategy by combining virus‐mimic surface topology and MAUS that can significantly improve the intratumor accumulation of NPs (Fig. 9a) [82]. The commercial dye molecule IR825 was conjugated with the magnetic virus‐mimic surface topological mesoporous silica Fe_3_O_4_@vSiO_2_ (abbreviated as MVSN‐IR825). The Fe_3_O_4_ is used as a T_2_ contrast agent of MRI and also can mediate the Fenton reaction to generate ROS. It was demonstrated that MVSN-IR825 exhibited the best timing of trimodal PTT/PDT/SDT, and provided an excellent cancer treatment effect in vitro experiment on subcutaneous and orthotopic PC‐3 xenograft tumor models (Fig. 9a).

###### Endogenous stimulus-responsiveness

Focal therapy has the advantage of low invasiveness and is suitable for the treatment of patients with early-stage PCa [170]. In order to enhance the efficacy of focal therapy, Hu et al. reported a prodrug nanoplatform with enhanced endocytosis, mitochondria-targeted and redox/enzyme-responsive behavior, where the cyanine dye IR806 was covalently conjugated to chondroitin sulfate (CS) via the redox-sensitive disulfide linkages to prepare an amphiphilic CS-ss-IR806 (CSR) conjugate (Fig. 9b) [80]. IR806 is the carboxyl derivative of IR780. It was reported that IR780 possesses excellent sonodynamic activity and mitochondrial-targeted capability [171]. CS, a natural biodegradable polysaccharide, can recognize the glycoprotein CD44 receptors overexpressed on tumor cells and can be decomposed by hyaluronidase-1 (Hyal-1). In vitro experiment and xenograft human PCa model, timing of trimodal PTT/PDT/SDT was observed with laser and ultrasound irradiation. The results suggested that CSR NPs were the most effective in suppressing tumor growth and killing tumor cells while achieving the trimodal synergism.

Cathepsin B, a lysosomal cysteine cathepsin, has been found to be hyper-secreted in the tumor environment of various solid tumors [172]. Hadi et al. synthesized a novel pH- and cathepsin B-responsive nanoparticle (HPNP) by self-assembly of poly(L-glutamic acid-L-tyrosine) co-polymer (PGA) with hematoporphyrin (Fig. 9c) [79]. Porphyrin analogs can respond to low-intensity ultrasound and generate ROS to inhibit tumor growth [18]. Low pH in the TME stimulates the secretion of cathepsin B from cancer cells, which mediated the breakdown of PGA and promoted the accumulation of nanoparticles in tumors. In in vitro experiments, the experimental group using HPNPs possessed higher cytotoxicity and more ROS yield on PCa cells (LNCaP and PC-3) than other groups. In xenograft LNCaP immunodeficient mice model, tumor volume decreased by 36% after 24 h of ultrasound treatment with HPNPs.

##### Combination therapy

###### Combination with chemotherapy

Synthesis of nanoparticles with imaging and therapeutic functions to realize the integration of diagnosis and treatment is a popular direction in the field of nanomedicine. Nanoparticles synthesized based on Ho^3+^, due to their properties of short electronic relaxation time (10 ~ 13 s) and large magnetic moment (≈10.0 µB), have been developed for MRI at ultrahigh fields [173]. For combining diagnosis and treatment in PCa, Wang et al. developed a kind of holmium-doped hollow silica nanospheres (HHSN), which was sequentially modified with chlorin e6, carboxyl poly(ethylene glycol) silane, and prostate stem cell antigen (PSCA) monoclonal antibody (Fig. 9d) [164]. The novel nanoparticles, HHSN-C/P-mAb, could target PCa cells due to the surface modification of PSCA monoclonal antibody. Inner cavity structure and Ho^3+^ doping of hollow silica endowed NPs with dual imaging capabilities of US imaging and MRI. Tirapazamine (TPZ), an important bioreductive hypoxia-selective cytotoxin, can be activated by intracellular reductases to generate cytotoxic ROS, leading to DNA fragmentation and cell killing in hypoxic environment [174]. While loading TPZ in the HHSN-C/P-mAb, the hollow silica spheres could be degraded in the acidic tumor environment, thereby triggering the release of TPZ at the tumor site. In addition, the oxygen-consuming SDT exacerbates tumor hypoxia and triggers the cytotoxicity of TPZ. What’s more, in PC-3 tumor-bearing nude mouse model, HHSN-C/P-mAb also showed excellent tumor targeting ability, US imaging and MRI ability (Fig. 9d). The synergistic therapy combining SDT and TPZ greatly demonstrated tumor elimination ability of TPZ@HHSN-C/P-mAb.

Non-photobleached, low-toxic and low-cost TiO_2_ nanoparticles have been reported to provide ultrasound-induced ROS generation efficiency [175]. Yuan et al. developed the MRI-guided PCa therapy based on the targeted drug nanosystem TiO_2_:Gd@DOX/FA activated by ultrasound [162]. Rare earth Gd were loaded with TiO_2_ for 2 main aims: (1) improving the ROS quantum yield of TiO_2_, and (2) endowing nanoparticles with the T_1_-weighted MRI ability. Folic acid (FA)-modified DOX were linked with TiO_2_:Gd via the hydrazone bond to improve the targeted uptake by tumor cells [176]. Due to the pH sensitivity of the hydrazone bond, DOX could achieve targeted release in acidic TME. The synthetic TiO_2_: Gd@DOX/FA NPs demonstrated high ROS yield, pH-dependent drug release sensitivity, T_1_-MRI contrast performance and excellent biocompatibility. Tumor cell elimination ability was observed in both vitro and in vivo experiments.

## Summary of strategies of enhanced PDT/SDT for urological cancers

As mentioned above, there are three main strategies for enhancing PDT/SDT for urological cancers through nanobiotechnology: (1) hypoxia alleviation; (2) improvement of targeted tumor cellular internalization; (3) combination therapy. Due to the hypoxia of TME and the oxygen-consuming nature of PDT/SDT, endowing nanoparticles with hypoxia-alleviating ability to improve efficacy is a general strategy applicable to all solid tumors and is not tumor-specific. In terms of improvement of targeted tumor cellular internalization, attaching targeting peptides to nanoparticles to target cell surface protein or enabling nanoparticles to release drugs in response to endogenous or exogenous stimulus can increase drug accumulation in lesions to a certain extent. Although these bioconjugated nanoparticles can achieve specific binding at the cellular level, they are still recognized by the immune system and cleared by the mononuclear phagocytic system in the human body [177]. With the further development of nanobiotechnology, imparting biomimetic coatings to nanoparticles has recently been used to overcome this limitation. Unique surface components of mammalian cells (e.g., erythrocytes, platelets and macrophages) or pathogens (e.g., viruses and bacteria) are most often used to impart biomimetic coatings to nanoparticles [178]. In combination therapy, combining PDT/SDT with clinically mature drugs is promising to help overcome resistance to these drugs or to induce expression of drug targets [48, 50].

It should be pointed out that this review only provides a preliminary classification of each paradigm by a single strategy, but in fact most of the paradigms we illustrated are a reasonable combination of multiple strategies, precisely because nanobiotechnology has unique advantages in integrating multiple functional components into a nanoplatform. PDT/SDT was not developed for specific cancer rather than pan-cancer until the further development of nanobiotechnology, which endowed nanoplatform-based PDT/SDT with targeting tumor-specific surface proteins, characteristic TME-responsiveness and integration of specific drugs.

## Current status and prospect of clinical translation

At present, the programs of PDT for urological cancers are emerging, and several relevant clinical trials have been carried out. The clinical trials of SDT for urological cancers have not yet been carried out and a large number of high-quality preclinical studies are still needed to be developed as a foundation. Herein, we mainly discuss the clinical translation status of PDT for urological cancers. Detailed information of PDT for urological cancers is shown in Table 4.

**Table 4:** Clinical trials of PDT for urological cancers

Photosensitizers	Conditions	Locations	Status	NCT number
TLD1433	NMIBC refractory to BCG	Canada	Phase 1 (C)	NCT03053635
	NMIBC refractory to BCG	United States; Canada	Phase 2 (R)	NCT03945162
Photofrin®	Superficial BC	United States	Phase 1/2 (C)	NCT00322699
Hexvix®	Intermediate or high-risk BC	NA	Phase 1 (C)	NCT01303991
Motexafin Lutetium	Adenocarcinoma of the prostate; recurrent PCa; stage I PCa; stage IIA PCa; stage IIB PCa	United States	Phase 1 (T)	NCT00005067
WST09	PCa	Canada	Phase 2 (C)	NCT00305929
	Recurrent or persistent localized carcinoma of the prostate following radiation therapy failure	Canada	Phase 2 (C)	NCT00308919
	PCa	Canada	Phase 2/3 (T)	NCT00312442
WST11	PCa	Canada; France; United Kingdom	Phase 2 (C)	NCT00707356
	PCa	United States	Phase 1/2 (C)	NCT00946881
	PCa	France; Netherlands; United Kingdom	Phase 2 (C)	NCT00975429
	PCa	Belgium; Finland; France; Germany; Italy; Netherlands; Spain; Sweden; Switzerland; United Kingdom	Phase 3 (C)	NCT01310894
	PCa	Mexico; Panama; Peru	Phase 3 (C)	NCT01875393
	Localized PCa	United States	Phase 2 (ANR)	NCT03315754
	Low Risk PCa	France	Phase 4 (T)	NCT03849365
	Localized PCa	United States	Phase 3 (W)	NCT04225299
	Upper tract urothelial carcinoma	United States	Phase 1 (ANR)	NCT03617003
	Renal cancer	United Kingdom	Phase 1/2 (T)	NCT01573156
	Transitional cell cancer of renal pelvis and ureter	United States; France	Phase 3 (R)	NCT04620239
Verteporfin	Recurrent PCa	United States; Canada; United Kingdom	Phase 1 (U)	NCT03067051

Data obtained from clinicaltrials.gov (Accessed on April 13, 2022)

*NA* Not available; *C* Completed; *R*: Recruiting; *T* Terminated; *ANR* active, not recruiting; *W* Withdrawn; *U* Unknown; *NMIBC* Non-muscle invasive bladder cancer; *BC* Bladder cancer; *PCa* Prostate cancer

Porfimer sodium (Photofrin®) is known as first-generation PS and was approved in Canada for the treatment of BC with PDT as early as 1993 [179]. However, shallow tissue penetration depth and prolonged skin phototoxicity limited its clinical application and thus drove the development of second- and third-generation PSs. Higher-purity second-generation PSs designed to reduce total drug dose to avoid adverse side effects when administered systemically are currently used in clinical trials for the treatment of urological cancers. 5-aminolaevulinic acid (ALA), a precursor of PS PpIX, accumulates preferentially in tumor tissues because it could induce up to tenfold in vivo PpIX fluorescence in malignant than in normal urothelium [180]. Recently, ALA-mediated PD had been approved in Japan for the management of NMIBC, and has been generally carried out to assist TURBT [181]. In a multicenter prospective trial in Russia, intraoperative ALA-PDT after TURBT reduced 1-year recurrence rate of NMIBC to 22%, superior to TURBT with traditional adjuvant therapy, such as chemotherapy and BCG [182], indicating ALA-PDT could be recommended for the treatment of patients with NMIBC. Moreover, several other PSs, such as Hexaminolevulinate (HAL, Hexvix®, Cysview®) [183], meta-tetra(hydroxyphenyl)chlorin (m-THPC, Foscan®) [184], and motexafin lutetium (Lu-Tex, Lutrin®) [185], were investigated in clinical trials for the treatment of BC (NCT01303991) or PCa (NCT00005067), but none of them have been clinically approved for photodynamic urological cancers therapy.

Encouragingly, WST11 (TOOKAD® soluble), a water-soluble derivative of WST09 (Padeliporfin, TOOKAD®), has been approved in Mexico, Israel and 31 countries of the EU for the treatment of low-risk PCa [186]. Vascular-targeted photodynamic therapy (VTP) using WST11 is a minimally invasive technique targeting tumor vessels rather than its parenchyma. Optical fibers within thin hollow needles are positioned in the prostate under ultrasound image guidance, and these fibers deliver 753 nm laser light that activates intravenously injected WST11 to generate thrombosis-causing ROS in blood vessels. The deprivation of oxygen and nutrients after vascular occlusion leads to cell death and tumor eradication [187]. A phase III trial done in 47 European university centers and community hospitals (NCT01310894) showed that a smaller proportion of patients with low-risk PCa in the VTP group had disease progression compared with patients in the active surveillance (one of the management strategies in men with low-risk PCa) group, and a higher proportion had negative prostate biopsy results at 24 months post treatment [188]. In addition, a phase III, single arm trial of WST11 in the treatment of upper tract urothelial carcinoma (UTUC) in the USA and France is enrolling patients (NCT04620239). TLD1433, a ruthenium polypyridine complex, has currently entered into a phase II trial for treating NMIBC with PDT in the USA and Canada (NCT03945162). The photophysical features of the ruthenium center extend the excited state lifetime of the transition metal complex and enable it to function through oxygen-independent type I reaction [189]. The development and translation process of TLD1433, which took 6 years from the bench to a clinical trial, has many highlights to reference for the scientists, clinicians, industrial partners and investors who are working on advancing PDT/SDT to clinical translation [190].

The inherent limitations of PDT/SDT for urological cancers can be summarized as: (1) drug resistance due to hypoxic TME; (2) low penetration depth of light into tissues; (3) low accumulation of photosensitizers/sonosensitizers in tumor areas; (4) poor understanding of SDT mechanisms; (5) inability to simultaneously treat metastatic tumors. It is a good idea to overcome these limitations via nanobiotechnology. As previously described, targeted third-generation photosensitizers or even multifunctional photosensitizers/sonosensitizers developed by nanobiotechnology have been reported in numerous preclinical studies, however, none of them have yet been investigated in clinical trials for the treatment of urological cancers. There are many reasons why translation from bench to bedside is so difficult for nanobiotechnology-assisted PDT/SDT. First, researchers focus on bottom-up synthesis of new compounds or nanoparticles, and then apply them to pan-cancer treatment study without a specific clinical indication. Second, there is no standardized parameter for the PDT/SDT process between different laboratories, and biological samples are inherently complex and uncontrollable, so the reproducibility of biological results in such studies is often problematic. Third, due to the separation of laboratory research and clinical needs, most studies did not evaluate newly developed photosensitizers/sonosensitizers together with clinically approved drugs. Fourth, most of the current clinical trials of PSs use PDT as adjuvant therapy, and the inclusion of patients with relapse or resistance to the original treatment led to sample selection bias.

Therefore, researchers should fully communicate with clinicians to clarify clinical needs, and then focus on specific cancer types and subtypes to synthesize and screen drugs. At the same time, standardization of in vitro assays is a prerequisite for ensuring the reproducibility of biological results. At least a standardized process should be used within a laboratory to quantitatively compare newly developed photosensitizers/sonosensitizers with clinically approved compounds. The key is to leverage the strengths of a multidisciplinary team and commercialization investments for developing a comprehensive suite of compounds/nanoparticles, medical devices and PDT/SDT protocols for target clinical indications. Researchers developing these compounds/nanoparticles cannot ignore the big picture, they should realize that without collaboration with clinicians, oncology biologists, manufacturers and investors, the value of these compounds will remain in concept but will not actually help patients.

## Conclusion

It has been nearly 50 years since PDT was firstly reported for the treatment of BC, and its efficacy in the treatment of tumors has been proven. WST11 has been approved in the EU, Mexico and Israel for the treatment of PCa. At the same time, there are several photosensitizers in the clinical trials for the treatment of urological cancers. However, PDT is not currently available as an alternative to traditional therapeutic modalities, such as surgery, radiotherapy and chemotherapy. SDT is a novel therapeutic modality with a similar working mode to PDT, which can overcome the defects of PDT (e.g., skin phototoxicity and shallow penetration depth), but its mechanism is still under investigation. There are currently no clinical trials of SDT approved for the treatment of urological cancers. Thanks to breakthroughs in nanobiotechnology, the arsenal of photo/sonosensitizers has expanded. Preclinical studies of nanobiotechnology-assisted PDT/SDT for urological cancers have exploded in recent years, and it has been demonstrated that by leveraging nanobiotechnology, the inherent limitations of PDT/SDT can be overcome and other therapeutic modalities can be combined with PDT/SDT. However, compared with the fiery preclinical researches of emerging photo/sonosensitizers, their clinical translation in the treatment of urological cancers is relatively slow, which means that scientists in academia should improve this photo/sonosensitizer-centric approach and collaborate with scientists in other fields, clinicians and investors. With the joint efforts of a multidisciplinary team, there is a bright future for nanobiotechnology-assisted PDT/SDT for urological cancer patients.

## Data Availability

Not applicable.

## Abbreviations

PDT: Photodynamic therapy
SDT: Sonodynamic therapy
ROS: Reactive oxygen species
PSs: Photosensitizers
UV/vis: Ultraviolet/visible
NIR: Near infrared
EU: European union
PCa: Prostate cancer
US: Ultrasound
HIFU: High-intensity focused ultrasound
ESWL: Extracorporeal shock wave lithotripsy
BC: Bladder cancer
TME: Tumor microenvironment
NMIBC: Non-muscle-invasive bladder cancer
MIBC: Muscle-invasive bladder cancer
TURBT: Transurethral resection of bladder tumor
HPD: Hematoporphyrin derivative
PD: Photodiagnosis
ALA: 5-Aminolevulinic acid
PpIX: Protoporphyrin IX
DFX: Deferoxamine
H_2_O_2_: Hydrogen peroxide
MRI: Magnetic resonance imaging
HSA: Human serum albumin
FCS: Fluorinated chitosan
MTP: Meso-tetra-4-pyridyl porphine
ssDNA: Single-stranded DNA
PDN: Porphyrin-DNA nanoparticles
GLUT1: Facilitative glucose transporter 1
PNP: PLZ4-nanoporphyrin
CA: Cholic acid
PEG: Polyethylene glycol
DOX: Doxorubicin
PDX: Patient-derived xenograft
NAC: Neoadjuvant combination chemotherapy
ZnPC: Zinc phthalocyanine
TNP: Thermal-responsive nanoparticle
PTT: Photothermal therapy
AIE: Aggregation-induced emission
AIEgens: Aggregation-induced emission luminogens
NIRFI: Near-infrared fluorescence imaging
DSP: Pt-2COOH
BSA: Bovine serum albumin
Ppa: Pheophorbide a
Erk: Extracellular signal-regulated kinase
Ce6: Chlorin e6
PTX: Paclitaxel
PCI: Photochemical internalization
HSP90: Heat shock protein 90
17-AAG: 17-Allylamino-17-demethoxygeldanamycin
HIF-1α: Hypoxia-inducible factor 1α
VEGF: Vascular endothelial growth factor
EGFR: Epidermal growth factor receptor
PIT: Photoimmunotherapy
IR700: IRDye700Dx
PET: Positron emission tomography
SCID: Severe combined immunodeficient
SIRPα: Signal regulatory protein-α
mAbs: Monoclonal antibodies
PSMA: Prostate-specific membrane antigen
BChI: Bacteriochlorophyll
NHS: N-hydroxysuccinimide
Pyro: Pyropheophorbide-a
cRGD: Cyclic cRGDfk
ABC: ATP-binding cassette
ABCB6: ATP-binding cassette subfamily B member 6
PEPT1: Peptide transporter 1
ABCG2: ATP-binding cassette superfamily G member 2
PA: Polyamine
PTS: Polyamine transport system
uPA: Urokinase plasminogen activation
uPAR: Urokinase plasminogen activation receptors
ECM: Extracellular matrix
HPLC: High performance liquid chromatography
MBs: Microbubbles
LFUS: Low-frequency ultrasound
PGL: Porphyrin-grafted lipid
GNs: Gold nanoparticles
PTX: Paclitaxel
TRPV6: Transient receptor potential cation channel subfamily V member 6
DTX: Docetaxel
GNS: Gold nanostars
PCSCs: Prostate cancer stem cells
CRPC: Castration resistant prostate cancer
PAI: Photoacoustic imaging
RCC: Renal cell carcinoma
ccRCC: Clear cell renal cell carcinoma
RP: Red phosphorus
MVs: Microvesicles
DCPy: (E)-4-(2-(7-(diphenylamino)-9-ethyl-9H-carbazol-2-yl) vinyl)-1-methylpyridin-1-ium hexafluorophosphate
FCS: Fluorinated chitosan
TCPP: Meso-tetra(4-carboxyphenyl)porphine
CAT: Catalase
TEER: Transepithelial electrical resistance
MAUS: Microbubble‐assisted ultrasound
CS: Chondroitin sulfate
Hyal-1: Hyaluronidase-1
PGA: Poly(L-glutamic acid-L-tyrosine) co-polymer
PSCA: Prostate stem cell antigen
TPZ: Tirapazamine
FA: Folic acid
VTP: Vascular-targeted photodynamic therapy
UTUC: Upper tract urothelial carcinoma
